# Substituent Effect in Histamine and Its Impact on Interactions with the G Protein-Coupled Human Receptor H_1_ Modelled by Quantum-Chemical Methods

**DOI:** 10.3390/molecules30183736

**Published:** 2025-09-15

**Authors:** Anna Jezuita, Małgorzata Makowska-Janusik, Krzysztof Ejsmont, Wojciech Marczak

**Affiliations:** 1 Faculty of Science and Technology, Jan Dlugosz University, Al. Armii Krajowej 13/15, 42-200 Czestochowa, Poland; a.jezuita@ujd.edu.pl (A.J.); m.makowska@ujd.edu.pl (M.M.-J.); 2 Department of Chemistry and Pharmacy, University of Opole, Oleska 48, 45-052 Opole, Poland; eismont@uni.opole.pl

**Keywords:** histamine, imidazole, imidazolium, human receptor H_1_, electronic structure, aromaticity, HOMA, cSAR, hydrogen bonds, DFT

## Abstract

Neutral and protonated histamine tautomers, mono-substituted with twelve functional groups, were studied theoretically as isolated molecules and complexes with the H_1_ receptor. Geometry and energy of tautomers were optimized using the DFT method with the B3LYP functional and the aug-cc-pVTZ basis set. The approach was based on the charge of the substituent active region (cSAR) parameters and the Harmonic Oscillator Model of Aromaticity (HOMA) indices. The cSAR parameters characterized the electron density better than the conventional Hammett’s constants *σ*. In general, the cSAR parameters correlate with other characteristics of the charge distribution, particularly those for substituents at the carbon atom in the ring adjacent to the side chain. Substituents at this atom affected the aromaticity less strongly than those located between two nitrogen atoms, which confirmed recent reports. Our results suggest that the 3H tautomer isomerizes into the 1H one after binding to the H_1_ receptor. Moreover, the electron structure of the molecule hydrogen-bonded to the receptor may significantly depend on the electron donor-acceptor properties of the substituent. The strong electron-accepting substituents, e.g., NO_2_, favor the imidazole configuration of the ring in the bonded molecule, while the strong electron-donating ones, e.g., NH_2_, promote the imidazolium one.

## 1. Introduction

Histamine plays a crucial role in the animal body’s defense mechanisms, including local immune responses and functions as a neurotransmitter [1]. The biological activity of histamine results mainly from its interaction with different types of specific G protein-coupled receptors (H_1_, H_2_, H_3_ and H_4_) in the cell membranes, which regulate various physiological processes [2]. Interactions of histamine with H_1_ lead to, e.g., cardiovascular responses, strong depressive action and allergic reactions. The activation of H_2_ receptors stimulates immunological reactions and the stomach secretions and regulates heart rhythm. Histamine-H_3_ interactions influence the nervous transmission. In turn, the binding of histamine to the H_4_ receptor affects the immunomodulatory and chemotoxic effects in various immune cells [1,3]. Substituents in the imidazole ring of the histamine molecule affect its affinity with receptors [4]. For instance, 2-methylhistamine is a weaker H_1_ agonist than histamine [2], while 5-methylhistamine is a selective H_4_ agonist [5]. Strong agonistic effects have been observed for histamine with halogen substituents [6,7].

**Scheme 1:**
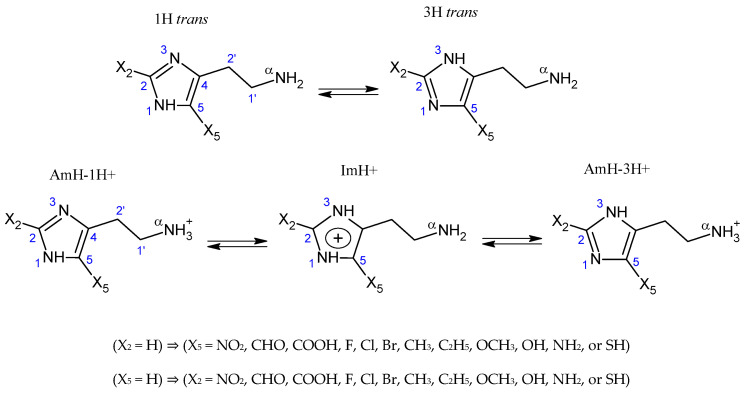
Monosubstituted histamine tautomers, neutral and monoprotonated, in the *trans* conformation.

Histamine, as a chemical substance, is a mixture of several molecular entities. It shows conformational isomerism [8,9]: the *trans* conformers occur in the solid state and aqueous solution, while the gauche ones predominate in the gas phase. Under physiological conditions, the dominant conformation of histamine is the *gauche*. However, studies on molecular docking of histamine indicate a key role of *trans* conformation in interaction with the receptor binding site [10]. Scheme 1 illustrates the histamine tautomers in neutral and cationic forms in *trans* conformations discussed in this work. As the *gauche* conformation involves intricate intramolecular interactions by closer proximity of the ^α^NH_2_ group to the imidazole ring, it will not be discussed further in this study.

Three nitrogen atoms in the molecule of histamine, which interact with active sites of the receptors, differ in basicity [11]. The N atom in the ring and the N-amino atom in the aliphatic side chain are basic, the latter one in particular. Contrary to those, the amino NH group of the ring is weakly acidic, just slightly more than the hydroxyl group in alcohols.

The neutral histamine exhibits a prototropic tautomerism consisting of a transfer of a proton from one nitrogen atom to the other in the imidazole ring, which is accompanied by the migration of π electrons, cf. 1H and 3H tautomers in Scheme 1. The tautomers differ in stability, dipole moment and solubility. 96% of histamine molecules form monoprotonated cations rather than remain neutral in aqueous solutions [12]. Protonation of the nitrogen atom in the imidazole ring (cf. ImH^+^ form in Scheme 1) requires a sufficiently acidic medium because p*K*a_1_ ≈ 5.8. At physiological pH, only the primary amine nitrogen atom of the side chain is protonated, p*K*a_2_ ≈ 9.4, cf. AmH-3H^+^ and AmH-1H^+^ forms in Scheme 1. Protonation of the nitrogen atom of the side chain is preferred in polar solvents such as chloroform, tetrahydrofuran, acetone, and water [13]. In the gas phase and non-polar solvents, the N atom in the ring is favorably protonated independently of the histamine conformation due to the high polarizability of the imidazole ring [14,15]. Substituents in the imidazole ring may also change the propensity of histamine to protonation, affecting its binding to the receptors [16].

The substituent constant, *σ*, is a conventional characteristic of the substituent effect (SE) suggested almost a century ago. It is given by the following empirical Hammett formula [17]:

(1) $$\sigma =\frac{1}{k}\log\frac{K}{K_{0}}$$

where *K*_0_ and *K* are the equilibrium constants for deprotonation reactions of benzoic acid and its substituted derivative, respectively, and *k* is the reaction rate constant, which depends on the reaction type and is assumed to be the same for the substituted and non-substituted benzoic acid. Usually, *σ* is reported for the *para* and *meta* substituents rather than the *ortho* one due to the disturbing steric effects for the latter. Thus, the stronger the acidity of the substituted derivative caused by the electron-withdrawing inductive effect and the negative mesomeric effect, the higher are the positive values of *σ*. Negative *σ* evidences the opposite effects.

The substituent constants are just rough characteristics of the SE. Despite this, the *σ* values correlated well with several quantum chemistry parameters, such as electrostatic potentials [18], HOMO/LUMO energies [19], substituent effect stabilization energy (SESE) [20], and the charge of the substituent active region (cSAR) [21]. Correlation with the charge located solely on the substituent was worse [21]. The energy decomposition analysis (EDA) also led to results coherent with *σ* [22]. However, the results of quantum chemical calculations provide deeper insight into the SE. In particular, the cSAR parameter shows a clear advantage over *σ*, as the latter does not depend on other substituents in the molecule and the environment [23,24].

Indeed, the quantum chemistry methods applied to biological compounds facilitate a quantitative assessment of the SE dependence on the environment, the transmitter type and the reaction site, intermolecular hydrogen bonds, and the intramolecular proximity effects [25,26,27]. The latter include intramolecular hydrogen bonds, steric hindrances, and the disturbed resonance effect [28].

In this work, we report the results of quantum chemical calculations of the substituent effect (SE) in histamine molecules presented in Scheme 1. To the best of our knowledge, histamine cations, which predominate in the biological environment, have never been studied in such a manner. We discuss the following specific issues:

- changes in the electrophilic and nucleophilic reaction centers of the histamine molecule due to substituents;
- substituent positions, tautomers, and proximity effects manifested in the SE;
- stability of substituted histamine molecules;
- molecular interactions with the H_1_ receptor;
- tautomerization of the substituted histamine molecules caused by binding to the receptor.

## 2. Results and Discussion

**Figure 1:**
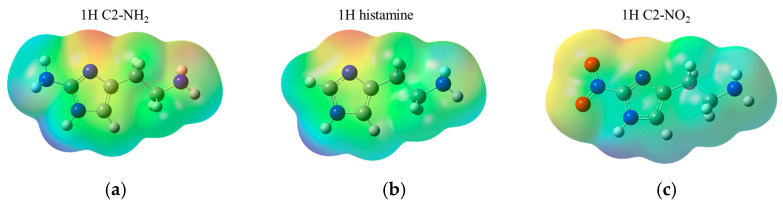
Electron density maps for the substituted and non-substituted histamine tautomer 1H trans. (**a**) X_2_ = NH_2_, (**b**) X_2_ = H, (**c**) X_2_ = NO_2_. X_5_ = H. The negative charge density is colored from blue (low density) to red (high density).

Charges of the substituent active region (cSAR) in the histamine molecule obtained in this work, together with the values of Hammett’s constants *σ*, are reported in Supplementary Table S1. Figure 1 illustrates the electron density in the molecule of histamine and its two derivatives with NH_2_ and NO_2_, i.e., the strongest electron-donating and electron-withdrawing substituents. The shifts in negative charge to and from the imidazole ring are evident. Similar effects occur in the other tautomers.

The electron-donating and electron-withdrawing groups were substituted for hydrogen atoms either at the C2 or C5 carbon atoms in the ring. Since *σ* depends on the substituent position in the ring, two series of these parameters were considered, those for the *meta* and *para* isomers, denoted as *σ*_m_ and *σ*_p_, respectively.

### 2.1. Substituent Effects Manifested in the cSAR(X) Values and Correlations with the Hammett’s Constants

We compared values of the constants *σ*_p_ and *σ*_m_ with the charge of the substituent active region (cSAR) for the neutral (3H and 1H) and three protonated (AmH-3H^+^, AmH-1H^+^ and ImH^+^) histamine tautomers shown in Scheme 1. Since the constant *σ* characterises differences between the electron density in the substituted and non-substituted aromatic ring, we calculated the charge shift caused by the substituent according to the following formula:

(2) ∆cSAR(X)=cSAR(H)−cSAR(X),

where X and H denote the substituent and hydrogen atom, respectively. The ΔcSAR(X) is positive for the electron-withdrawing substituents and negative for the electron-donating ones. Thus, the sign of ΔcSAR(X) would be the same as that of the *σ* value for any substituent if the two methods assess the substituent effect consistently. A formula analogous to Equation (2) was also applied to other cSAR parameters.

First, we analysed correlations of the cSAR(X_2_) values, i.e., these for the substituents X_2_, with the *σ*_p_ constants. Seven out of twelve substituents show positive values of *σ*_p_ and can be ranked according to the decreasing electron-withdrawing ability as follows: NO_2_, COOH, CHO, Br, Cl, SH, and F. Non-substituted histamine molecule is a reference system with *σ*_p_ = 0. The remaining five substituents: C_2_H_5_, CH_3_, OCH_3_, OH, and NH_2_ do not withdraw electrons from the ring.

**Figure 2:**
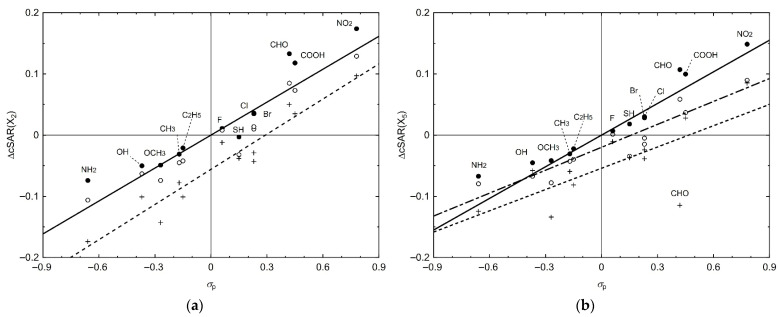
Correlation of Hammett’s constants *σ*_p_ with the ΔcSAR shifts (Equation (2)) for the histamine derivatives with various substituents in the imidazole (or imidazolium) ring, cf. Scheme 1. Points—calculation results; lines—fitted Equation (8). (**a**) Substituents in position X_2_: ●—3H (neutral), ○—AmH-3H^+^ (protonated), +—ImH^+^ (protonated with the positively charged ring). Solid line—equation with *b* = 0, common for 1H, 3H, AmH-1H^+^, and AmH-3H^+^; dashed line—equation with *b* ≠ 1 for ImH^+^. (**b**) Substituents in position X_5_; symbols as in (**a**). Solid line—Equation (8) with *b* = 0 for 1H and 3H (neutral); long dash short dash line—equation with *b* ≠ 1 for AmH-1H^+^ and AmH-3H^+^ (protonated); dashed line—equation with *b* ≠ 1 for ImH^+^. The ΔcSAR values for 1H trans and AmH-1H^+^ did not differ significantly from those plotted for 3H trans and AmH-3H^+^; thus, the respective points were omitted for picture clarity.

The ΔcSAR(X_2_) parameters are well-correlated with *σ*_p_ for the four tautomers of histamine: the neutral 3H and 1H and protonated AmH-3H^+^ and AmH-1H^+^. The simple regression Equation (8) with *y* = ΔcSAR(X_2_) and *x* = *σ*_p_ were fitted to the four sets of data. The *t*-test evidenced that the four intercept constants *b* did not differ from 0, and the regression coefficients *a* were equal at the significance level of 0.05. Indeed, the intercept constant in any correlation equation should be equal to 0 because no charge shift occurs for the hydrogen atom (i.e., in the non-substituted ring) by definition. Then, the calculations were repeated with the single linear Equation (8) for the 48 pairs of data, i.e., twelve data points for each of the four tautomers. Again, the intercept constant *b* proved to be equal to 0 based on the results of the *t*-test at the significance level of 0.21. The latter value is much higher than the usually accepted critical value of 0.05. Eventually, the linear Equation (8) with the intercept constant *b* set to zero was fitted to the 48 pairs of the *σ*_p_ and ΔcSAR(X_2_) values. The fitting coefficient *a* and its standard error obtained by the least-squares method, along with the coefficient of determination *R*^2^, are reported in Table 1. The slope, *a*, lies in the interval from 0.16 to 0.20 at the confidence level of 0.95. Figure 2a illustrates the good quality of the fit. For the neutral 3H and 1H tautomers, the substituents can be arranged in the following order: NO_2_, CHO, COOH, Br, Cl, F, (H), SH, C_2_H_5_, CH_3_, OCH_3_, OH, and NH_2_ according to the decreasing electron-withdrawing ability characterized by the value of cSAR(X_2_). The underlining marks the substituents which switched positions in comparison with a similar arrangement based on the *σ*_p_ values. All substituents except SH were classified into the same electron-donating or electron-withdrawing group by the two parameters. SH is a weak electron-donating substituent according to the cSAR(SH) value, while positive *σ*_p_ suggests that it withdraws electrons more strongly than the F substituent does. However, this discrepancy is rather small, as illustrated by the SH point close to that with the (0,0) coordinates in Figure 2a. It can be summarized that the halogens and SH as substituents virtually do not influence the charge distribution because of the negative inductive and positive mesomeric effects cancelling each other out.

The *σ*_p_ and ΔcSAR(X_2_) for the protonated ImH^+^ tautomer do not fulfil Equation (8), which is illustrated by the pronounced downward shift in the regression line in Figure 2a. In this case, the *t*-testing evidenced the intercept coefficient *b* ≠ 0 in Equation (8) (cf. Table 1). Moreover, four substituents, SH, F, Cl and Br, do not withdraw electrons from the imidazolium ring, ΔcSAR(X_2_) < 0, despite being classified as modest electron acceptors according to *σ*_p_ > 0. As could be expected, the positive charge impedes the withdrawal of the electrons from the imidazolium ring. Nevertheless, the correlation of *σ*_p_ with ΔcSAR(X_2_) for IMH^+^ is preserved.

An attempt to correlate Hammett’s constants for *meta* isomers (*σ*_m_) with the values of ΔcSAR(X_2_) gave less satisfactory results, as illustrated by the calculation results in Table 1. Three two-parameter equations analogous to Equation (8) were necessary to describe the correlations for the five tautomers: one for 3H and 1H, one for the protonated AmH-1H^+^ and AmH-3H^+^ and one for the ImH^+^ tautomer. In all three cases, the correlation lines are shifted downwards in comparison with the previous ones, which is manifested in the negative values of the *b* coefficients.

A similar procedure of selecting and fitting correlation equations was applied to the results for the X_5_ substituents. Equation (8) with *b* = 0 was suitable only for the combined dataset 1H and 3H. For the other datasets, Equation (8) with negative intercept constants were necessary. The weakest correlations are between the ΔcSAR(X_5_) values and Hammett’s constants for substituents in the *meta* position, *σ*_m_. In all cases, the two-parameter correlation Equation (8) was applied, and the determination coefficients were lower than previously. The coefficients of the correlation equations are reported in Table 1, and the graphs are plotted in Figure 2b.

This part of the study leads to the conclusion that Hammett’s constants for para isomers predict similar electron shifts as the ΔcSAR(X_2_) parameters for neutral molecules of histamine and ions with positive charge on the nitrogen atoms of the side chain. The agreement is worse for the ΔcSAR(X_5_). Hammett’s constants for *meta* isomers are less suitable for the prediction of the substituent effect in the molecules of histamine derivatives. In general, the cSAR approach shows a substantial advantage over that based on Hammett’s constants. This is evident for the ionic forms of histamine, those with the positively charged imidazolium ring in particular. Substituents classified as weakly withdrawing electrons according to the *σ* values may act rather as weak electron donors, e.g., F, SH, Cl and Br. The negative intercept coefficients in Equation (8) manifested as downward shifts in the regression lines plotted in Figure 2, evidence that Hammett’s constant approach led to slightly overestimated electron-withdrawal ability of the substituents.

### 2.2. Substituent Effect on the Aliphatic Chain of Histamine

Substituents affect the reaction sites in histamine derivatives, i.e., the aliphatic NH_2_ group (^α^NH_2_) in the side-chain and N atoms in the imidazole ring. This phenomenon may be called the classical substituent effect (SE). Changes in electron density on the aliphatic chain, including the ^α^NH_2_ group and the *ipso* carbon atom, are manifested in the cSAR(aliph) parameter (cf. Section 3). In this respect, the cSAR(aliph) parameter provides deeper insight into the SE than the cSAR(NH_2_), which deals only with the local electron structure of the NH_2_ group and the adjacent carbon atom of the chain. For the same reason, the ranges of cSAR(aliph) values are wider than those of the respective cSAR(NH_2_) ones (Table S1).

**Figure 3:**
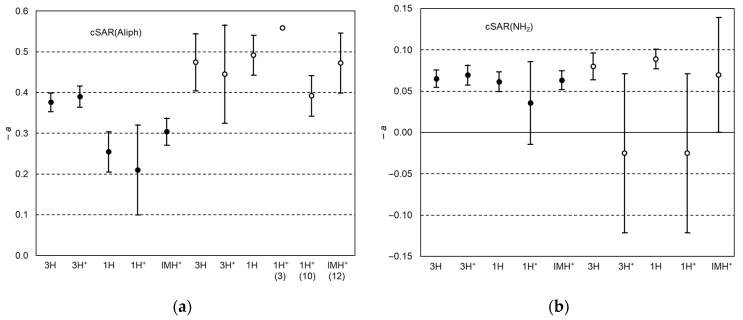
Negative slopes of the *y* = *a* ∙ cSAR(X) + *b* regression lines and their ranges at the confidence level of 0.95 for the histamine tautomers: (**a**) *y* = cSAR(aliph), (**b**) *y* = cSAR(NH_2_). Filled symbols—the substituents at the C2 atom of the ring, empty symbols—the substituents at the C5 atom (cf. Scheme 1). The number of data points in each dataset is 13; otherwise, the number is given in parentheses.

An effect of a substituent in the histamine ring on the electron density at the side chain is illustrated in Figure 1. Indeed, the side-chain electron density is sensitive to the substituents in the ring, which is manifested in the slope of the simple regression line cSAR(aliph) vs. cSAR(X). The parameters of the regression Equation (8) and the respective graphs are reported in the Supplementary Table S2 and Figure S1. Figure 3 illustrates a comparison of the slopes and their ranges assessed from the standard errors at the confidence level of 0.95. The determination coefficients *R*^2^ varied from 0.614 for the dataset C2-AmH-1H^+^ to 0.992 for C2-3H (neutral). For two datasets, the correlations were disturbed by outliers: the results for NO_2_, COOH, and CHO in C5-AmH-1H^+^ and CHO in C5-ImH^+^. Thus, the calculations were repeated for the dataset C5-AmH-1H^+^ split into two subsets: a subset without the outliers and a second subset with the data for NO_2_, COOH, and CHO only. A statistical analysis was impossible for the latter because of the insufficient number of data points. An illustration is given in Figure 3a. The calculations were also repeated for the dataset C5-ImH^+^ without the outlier CHO.

A similar analysis suggests that the substituents in the ring changed the local electron density on the NH_2_ group and the adjacent carbon atom less than on the whole side chain. This idea is supported by rather small values of the regression coefficients *a* of the cSAR(NH_2_) vs. cSAR(X) correlation Equation (8), which is illustrated in Figure 3b. Statistically significant correlations do not occur between cSAR(NH_2_) and cSAR(X) for C2-AmH-1H^+^ (*R*^2^ = 0.185), C5-AmH-1H^+^ (*R*^2^ = 0.411), and C5-AmH-3H^+^ (*R*^2^ = 0.029), i.e., *a* = 0 for these datasets at the confidence level 0.95. For the C5-ImH^+^ dataset, the coefficient *a* differs from 0 only slightly, −0.139 < *a* < −0.001 at the 0.95 confidence level, and *R*^2^ = 0.309. The maximum *R*^2^ = 0.961 for C5-1H. All the regression parameters and graphs are reported in the Supplementary Table S2 and Figure S2.

The results of this part of the study confirm that the substitution of an electron-donating group in the ring causes an increase in the electron density at the side chain of the histamine molecule. This phenomenon occurs in all histamine tautomers, independently of the substituent position, C2 or C5. However, the charge shift due to the substituent weakens along with the side chain length. Consequently, the electron density at the terminal ^α^NH_2_ group is less sensitive to the substituent properties than that at the whole side chain. The screening effect of the chain is pronounced for the cationic tautomers in particular, except for the AmH-3H^+^ substituted at the C2 atom.

A detailed discussion of the substituent effect is in the next two sub-sections.

#### 2.2.1. Neutral Forms of Histamine Derivatives

In C2 substituted neutral 3H and 1H tautomers, the correlation between cSAR(aliph) and cSAR(X) (cf. Equation (8)) is strong and negative, *R*^2^ = 0.992 and *R*^2^ = 0.921, respectively. The substituent effect is ca. 1.5 times stronger in 3H than in 1H tautomers, as suggested by the values of the slopes of the regression lines: *a*(C2-3H) = −0.37 and *a*(C2-1H) = −0.25 (see Figure S1). This increased sensitivity in the 3H tautomer is due to the resonance effect being weaker than in the 1H one. Indeed, the conjugation of the lone electron pair on the N1 atom (such as in 3H) with the π-system of the ring is less effective in comparison with that of the pair on the N3 atom (as in 1H). Consequently, the inductive effect is more pronounced in the 3H tautomer.

A substituent at the C5 atom adjacent to the side chain disturbs the electron density at the chain more effectively than that at the C2. The reinforcement can be dubbed a “proximity effect”. It is manifested in the ca. 1.5 times larger negative slopes of the cSAR(aliph) vs. cSAR(X) regression lines (Equation (8)). The slopes are –0.47 and –0.49, for C5-3H and C5-1H, respectively, and they are statistically equal (*p* = 0.669). These numbers characterize molecules with optimized geometry (cf. Section 3), with the aliphatic side chain bent out of the imidazole ring plane. Those for co-planar conformations (non-optimal ones) differ only slightly, e.g., *a* = −0.46 for C5-3H co-planar. A similar was observed for the 1H tautomer. Thus, the conformation of the aliphatic chain of the histamine molecule does not significantly affect its interactions with substituents. This is another argument for the inductive effect diminishing with the distance, apart from that derived from the analysis of the cSAR(NH_2_)–cSAR(X) correlations.

Similar qualitative conclusions can be drawn from the cSAR(NH_2_) vs. cSAR(X) correlations, albeit the slopes of the regression lines were almost one order of magnitude smaller than those for cSAR(aliph) ones. The rather weak effect of the C2 (*meta*) substituent on cSAR(NH_2_) is because the aliphatic chain interacts with the imidazole system through inductive effects along the sigma bonds, without participating in π-electron delocalization. The proximity effect manifests itself in the slightly stronger influence of the C5 (*ortho*) substituent on the ^α^NH_2_ electron density.

#### 2.2.2. Cationic Forms of Histamine Derivatives

Cationic forms of histamine derivatives contain either a protonated nitrogen atom in the imidazolium ring (as the second NH group) or in the aliphatic chain (as NH_3_^+^). The imidazolium ring may electrostatically interact with the active site of the receptor. In turn, electron-accepting NH_3_^+^ is a better H-bond donor with amino acids. The question was whether the SE in protonated tautomers differed from that in the neutral ones.

The cSAR(aliph) parameters for cationic tautomers are well-correlated with the cSAR(X) ones, which is manifested in the determination coefficients *R*^2^ > 0.61. The regression lines are plotted in Figure S1, and the details are presented in Table S2. The slopes of the correlation Equation (8) for the cationic forms, including ImH^+^, did not differ significantly from those for the respective neutral ones, which is illustrated by the overlapping error bars in Figure 3. Albeit the cSAR(aliph) increases in the order 1H (or 3H) neutral–ImH^+^–AmH-1H^+^ (or 3H^+^) which reflects the gradual decrease in the negative charge density at the terminal ^α^NH_2_ (eventually ^α^NH_3_^+^) group, the electron density at the hydrocarbon chain is influenced by a substituent in the ring in the same manner for the neutral and ionic tautomers.

**Figure 4:**
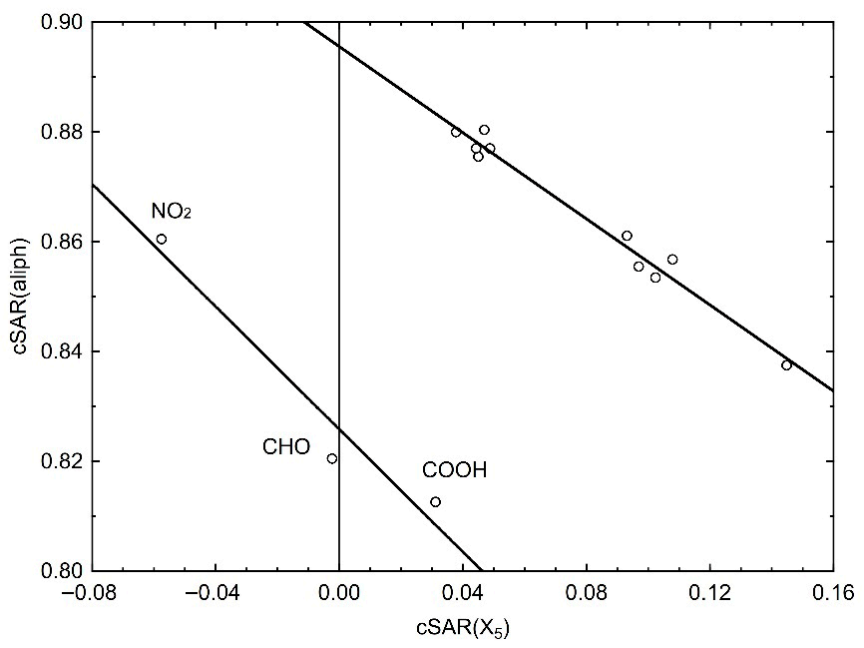
Simple regression lines for the correlations of cSAR(X_5_) parameters with cSAR(aliph) for the AmH-1H^+^ tautomer. Upper line and the neighboring points—a subset of the calculation results for ten substituents out of thirteen studied; lower line—such a result for the three remaining substituents: NO_2_, CHO, and COOH.

**Figure 5:**
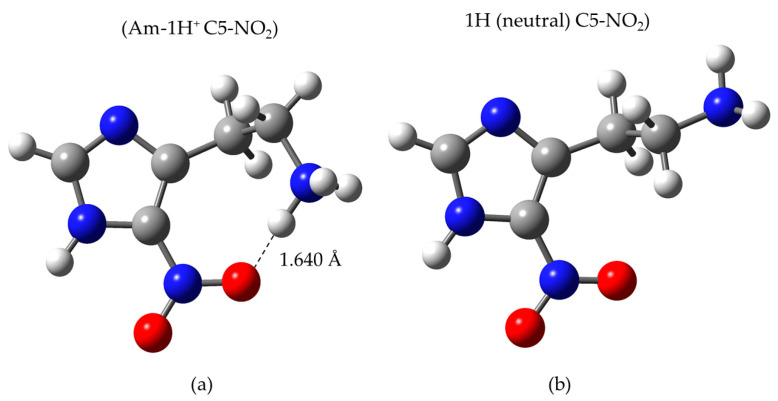
Intramolecular hydrogen bond in the optimized structure of the cationic tautomer of histamine substituted with an electron-withdrawing group in the *ortho* position towards the chain (**a**), and the corresponding optimized structure of the neutral 1H tautomer (**b**). Atoms: carbon—dark grey, hydrogen—light gray, nitrogen—blue, oxygen—red.

The only result that requires a comment here is that for the AmH-1H^+^ tautomer with a substituent at the C5 atom in the ring, cf. Figure 4. The correlation Equation (8) for the group of ten substituents, from the strong electron-donating NH_2_ group to the halogen atoms, shows the slope *a* is only slightly smaller than that of the respective equation for the neutral 1H tautomer. Thus, this result for C5-AmH-1H^+^ shows similar regularity as that for the following three pairs: C2-3H (neutral) and C2-AmH-3H^+^, C2-1H (neutral) and C2-AmH-1H^+^, and C5-3H (neutral) and C5-AmH-3H^+^. A reversed relationship is shown for the electron-accepting substituents NO_2_, COOH, and CHO in C5-AmH-1H^+^. Here, the slope *a* is slightly bigger than that for C5-1H (neutral). However, the number of data points is too small for a reasonable statistical analysis. We may just speculate that the reversal is due to intramolecular interactions between the oxygen atom of the substituent and the electron-accepting NH_3_^+^ group, as shown in Figure 5. This peculiarity was not observed for the cation structure with the aliphatic chain coplanar to the imidazole ring. In the latter case, the coefficient of determination *R*^2^ = 0.96 evidences a very high negative correlation between cSAR(aliph) and cSAR(X_5_).

### 2.3. Substituent Effect on N Atoms of the Ring

**Figure 6:**
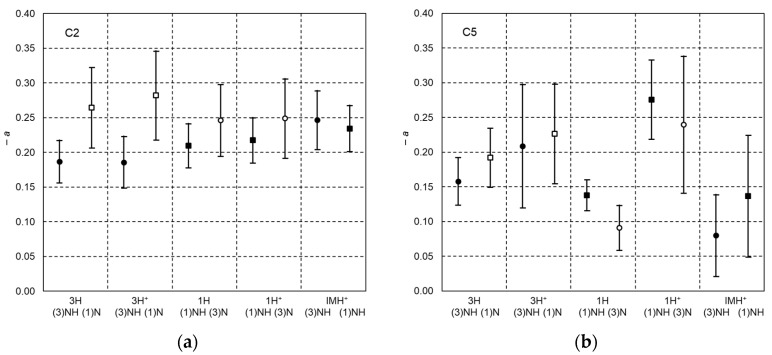
T Negative slopes of the electron density vs. cSAR(X) regression lines, *q* = *a* ∙ cSAR(X) + *b*, and their ranges at the confidence level of 0.95 for the histamine tautomers: (**a**) substituents at the carbon atom C2, (**b**) substituents at the carbon atom C5. Filled symbols—*q* at the NH group, empty symbols—*q* at the non-protonated N atom; circles—nitrogen atom N(3), squares—nitrogen atom N(1). Tautomers in their optimum geometry.

A molecule of histamine also interacts with the amino acid receptors through the N atom and NH group in the imidazole ring [29]. In this section, we analyze changes in the electron density at these reaction centers caused by substituents. The calculated electron densities on the N atom and NH group are reported in Table S3. The regression parameters of Equation (8) for the electron density *q* vs. cSAR(X) are collected in Table S4. Figure 6 illustrates a comparison of the slopes *a*.

Substituents at the C2 atom influence the electron density at the N atoms of the ring slightly more strongly than those at C5, which results in steeper regression lines for the C2 tautomers than for the C5 ones. Only in two cases out of ten, the relationship seems to be reversed, i.e., the steeper lines for C5: for the NH(1) group in AmH-1H^+^ tautomer, and the NH(3) group in 3H(neutral) one. However, the differences are small indeed, as illustrated by the overlapping error bars in Figure 6.

In particular, the electron density at the non-protonated N atoms of the ring is almost equally sensitive to the substituents at the C2 atom, regardless of the tautomer (cf. Figure 6a). Less sensitive is that at the NH group. The difference in sensitivities is greater for the 3H tautomers (neutral and charged alike) than for the 1H ones. This probably results from the stronger resonance effect in the 1H than in the 3H tautomer, due to more effective conjugation of the lone electron pair at the N3 atom with the π-electron system. In the IMH^+^ tautomer, the charged imidazolium ring enhances the SE on the electron density at the NH groups. Since the two groups are in the *ortho* position towards the substituent, the magnitudes of the SE are virtually the same for one group as for another.

The picture is different for the histamine molecules substituted at the C5 atom of the ring because of their lower symmetry—two nitrogen atoms are located *ortho* and *meta* towards the substituent. In general, the substituents more strongly influence the electron density at the *ortho* nitrogen atom than at the *meta* one, regardless the atoms are protonated or not, in neutral or cationic tautomer, cf. Figure 6b. However, the differences are small and even statistically insignificant for the C5-3H tautomers, neutral and cationic, and for cationic C5-AmH-1H^+^. The protonation of the ^α^NH_2_ group of the chain significantly enhances the SE on the ring nitrogen reaction centres in the C5-1H tautomers, while only slightly in the C5-3H ones. Contrary to that in symmetrical C2 tautomers, the SE on the electron density at the NH groups in the C5-ImH^+^ ring depends on their location towards the substituent. The SE for the *ortho* N can be assessed as 1.7 times stronger than that for the *meta* N, based on the ratio of respective slopes of the regression lines.

### 2.4. Effect of Substituents on the Aromaticity of Histamine Derivatives

Strongly electronegative N atoms withdraw electrons from the imidazole ring, affecting its aromaticity. Substituents in the ring influence the π-electron delocalization. For imidazole, the geometric HOMA (Harmonic Oscillator Model of Aromaticity) index is equal to 0.884, while it is 0.873 for 3H and 0.879 for 1H tautomers of histamine. These slightly lower HOMA indices suggest that the NH_2_ of the aliphatic chain in the histamine molecule influences the sigma electron structure in the ring due to a weak inductive interaction. Protonation of the ^α^NH_2_ group increases the aromatic character of the histamine ring, leading to the HOMA values of 0.889 and 0.894 for the AmH-3H^+^ and AmH-1H^+^ histamine tautomers, respectively. In turn, protonation of the ring (ImH^+^) enhances the localization of π-electrons, as evidenced by the lower HOMA value of 0.863. Wieczorkiewicz et al. suggested recently that the aromaticity of the imidazole molecules substituted at C4 and C5 atoms is 3.4 times less sensitive to the SE than that of C2-substituted derivatives [30]. A substituent adjacent to the NH group in the ring affects the aromaticity more effectively than those at other atoms. The effects of strong electron-withdrawing substituents are particularly pronounced.

**Figure 7:**
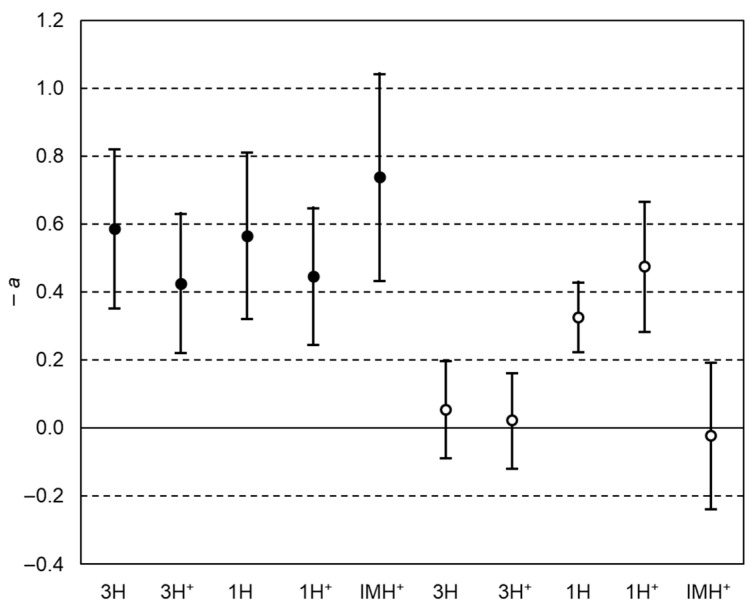
Negative slopes of the HOMA vs. cSAR(X) regression lines, HOMA = *a* ∙ cSAR(X) + *b*, and their ranges at the confidence level of 0.95 for the histamine tautomers: filled symbols—substituents at the carbon atom C2, empty symbols—substituents at the carbon atom C5.

We focused this part of the study on correlations of HOMA indices with cSAR(X) parameters for the C2 and C5 substituted histamine molecules. The values of the HOMA indices, coefficients of the correlation Equation (8), and determination coefficients are reported in Supplementary Table S6. Figure 7 illustrates a comparison of the slopes of the simple regression Equation (8).

The correlations are statistically insignificant for C5-substituted tautomers 3H(neutral), AmH-3H^+^ and ImH^+^, resulting in the slope coefficients of the regression lines equal to 0 at the confidence level of 0.95. For the other tautomers, negative correlations suggest that the stronger the electron-donating effect of the substituent, the less aromatic is the imidazole (or imidazolium) ring due to the weaker delocalization of the π electrons and the enhanced substituent effect. Protonation of the ^α^NH_2_ group slightly weakens this effect in the C2-substituted tautomers. The SE is enhanced for the C2 imidazolium ring.

**Figure 8:**
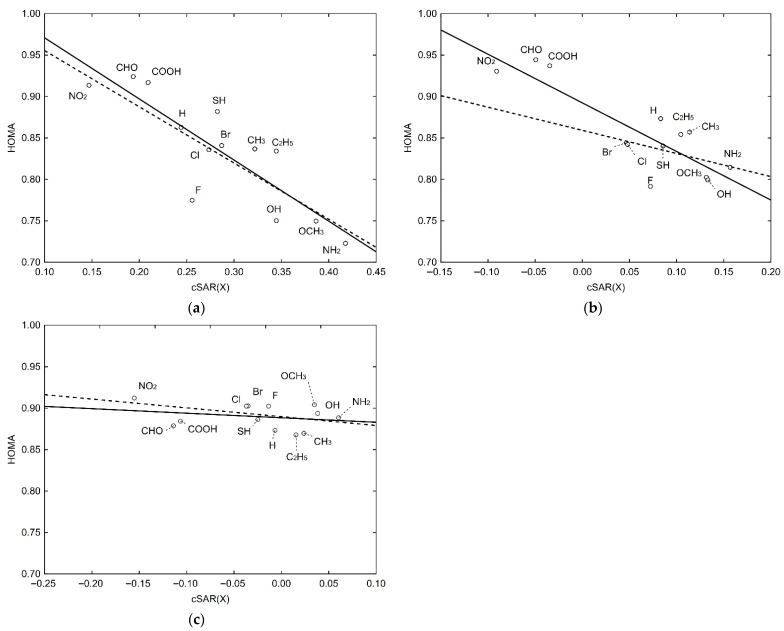
Scatterplots of HOMA vs. cSAR(X) with fitted regression lines HOMA = *a* ∙ cSAR(X) + *b* for the histamine tautomers: (**a**) C2-ImH^+^, *R*^2^ = 0.721; (**b**) C2-3H(neutral), *R*^2^ = 0.735; (**c**) C5-3H(neutral), *R*^2^ = 0.060. Solid lines—least squares fit to all 13 data points; dashed lines—least squares fit to 10 data points, those for NO_2_, CHO, and COOH excluded.

The scatter plots reported in Figure 8 illustrate three types of the cSAR(X) vs. HOMA relationships observed for the histamine tautomers. For simplicity, we shall name the types (a), (b) and (c) that correspond with the symbols of the three plots in Figure 8.

The scatter of type (a), exemplified by the results for C2-ImH^+^, is approximately uniform along the regression line. Consequently, the two regression lines, one fitted to all 13 data pairs and the second fitted to 10 data pairs (a subset without the data for the electron-withdrawing NO_2_, CHO and COOH substituents), nearly overlap one another. The determination coefficient for the first one is fairly high, 0.721. The results for C2-AmH-1H^+^ and C2-AmH-3H^+^ follow this pattern, although a gap occurs between the data points for NO_2_, CHO, and COOH substituents and those for the ten remaining ones. Here, too, the quality of fit is acceptable: *R*^2^ = 0.684 and *R*^2^ = 0.654, respectively.

The gap is wider in type (b), represented by the results for C2-3H(neutral), C2-1H(neutral), C5-1H(neutral), and C5-1H^+^ tautomers. The determination coefficients in this group, equal to 0.735, 0.701, 0.816, and 0.732, respectively, are even higher than those for the type (a) correlations. However, this hardly evidences a “better quality” of the fits but rather results from the wide gap between the two groups of data: one of those for the three electron-withdrawing substituents (NO_2_, CHO and COOH) and the second with the data for the remaining ten substituents. The slope coefficients of the regression lines fitted to the latter datasets do not differ from zero at the confidence level of 0.95. Indeed, such a line plotted in Figure 8b is much less steep than that fitted to all 13 data points. Thus, the HOMA indices can be assessed just roughly from the cSAR(X) parameters using the correlation Equation (8) for these systems.

The results for C5-3H(neutral), C5-3H^+^, and C5-ImH^+^ tautomers belong to type (c); the respective determination coefficients are 0.061, 0.028, and 0.005. Here, the HOMA indices are close to one another, and their values lie in the range from 0.850 to 0.930. The HOMA indices are not correlated with the cSAR(X) coefficients of these tautomers.

We may conclude that the arrangement of the π electrons in the ring is more sensitive to the properties of substituents at the C2 atom of the tautomers than the C5 one (see Figure 7). Substituents at the C5 atom disrupt the π electrons provided that only the adjacent N3 atom of the ring is protonated. The latter effect seems to be somewhat smaller than those in the respective C2 tautomers. Because of the substantial uncertainties of the determined coefficients *a* (cf. Figure 7), the conclusions should be drawn with due care. We just point out several regularities and suggest rudimentary explanations.

**Figure 9:**
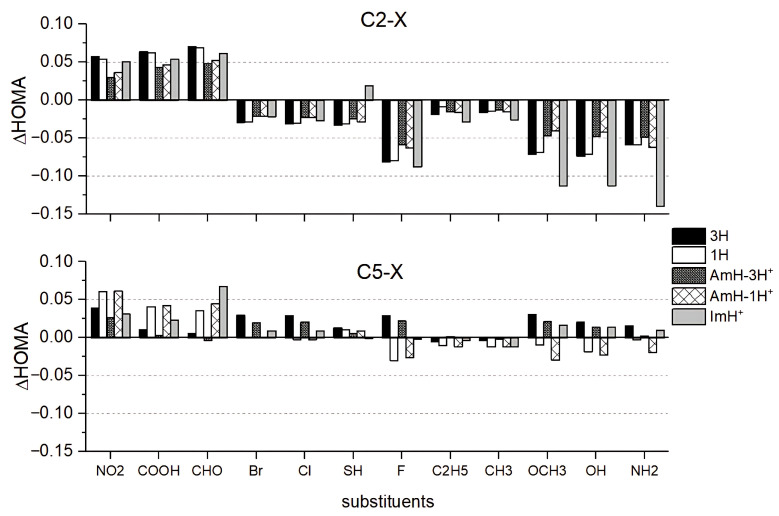
Aromaticity of the C2- and C5-substituted histamine tautomers related to those of respective unsubstituted molecules: ∆HOMA = HOMA(X) − HOMA(H).

Changes in the aromaticity due to the substitution at C2 are of similar magnitude for 3H and 1H tautomers in neutral and cationic forms. Thus, the tautomer structure has little effect on the aromaticity changes due to the substitution. This is illustrated in Figure 9. Slightly smaller changes in aromaticity due to the SE, manifested in the ΔHOMA values, occur in the C2-AmH+ than in the respective neutral tautomers. The difference is probably due to the inductive interactions between the electron-withdrawing moieties: the N atoms of the ring and the NH_3_^+^ group, which stabilizes the π electrons of the ring and, consequently, the aromaticity is less prone to changes.

Protonation of the N atom in the ring disrupts the arrangement of π-electrons. This is manifested in breaking Hückel’s rule, as the number of π-electrons in the ring is no longer equal to 4*N* + 2. A slightly stronger influence of the C2 substituents on the HOMA indices of the imidazolium tautomer in comparison with those of the imidazole ones (cf. Figure 7) suggests a reduced π conjugation in the former. This explanation can be supported by an analysis of the π-electrons delocalization due to the SE, illustrated in Figure 9. Indeed, the delocalization in the C2-ImH^+^ tautomer with the electron-donating substituents, e.g., NH_2_, OH, and OCH_3_, is more pronounced than in the other tautomers. It suggests the ring be the electron-accepting center, interacting more effectively with the ED substituents.

Substitution at the C5 atom influences the aromaticity much less than that at the C2, resulting in lower variability in the values of the HOMA index (cf. Figure 9). This is due to the weaker resonance effect in the C5 tautomers than in the C2 ones. However, contrary to the case of C2-substituted tautomers, the C5 tautomer structure is crucial for the substitution effect on aromaticity. Thus, the C5-1H(neutral) and C5-AmH-1H^+^ tautomers are nearly equally sensitive to the SE as their C2 counterparts. Substituents hardly change the aromaticity of C5-3H(neutral), C5-3H^+^, and C5-ImH^+^ tautomers, because they are adjacent only to the N1 atom rather than to the two N1 and N3 atoms. The N1 lone pair of electrons is not conjugated to the π-system, which weakens the resonance effect in the ring.

### 2.5. Molecular Characteristics of the Histamine Derivatives and Biological Activity

Substituted histamine derivatives with the tautomerization equilibrium substantially shifted towards one side are weaker agonists of the H_2_ receptors than unsubstituted histamine [31]. The methyl group at the C5 atom of the imidazole ring provides selectivity towards the H_2_ receptors and causes almost 50% loss of agonistic efficacy [31]. Another example of weaker agonists is the NO_2_-C5-substituted histamine [31].

Table 2 presents a compilation of several theoretical indices of the histamine derivatives obtained in this work and the characteristics of biological activity.

**Figure 10:**
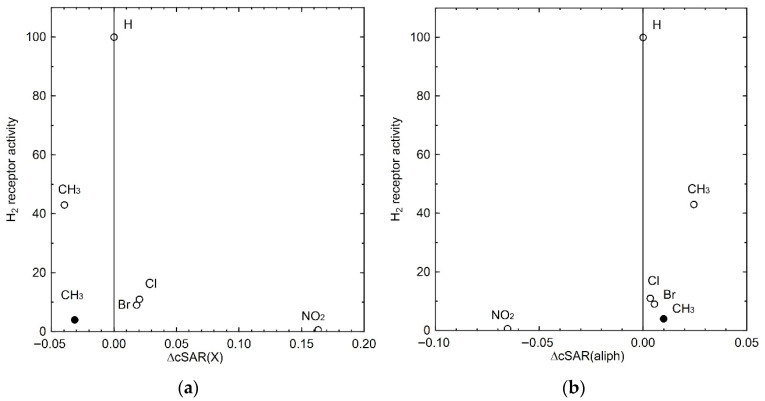
Reduction in the H_2_ receptor activity caused by substituents of various cSAR(X) (**a**) and cSAR(aliph) (**b**) parameters in neutral histamine tautomers 1H. Substituents at the carbon atom C5 (○) and the CH_3_ at C2 (●).

The scarce data on the H_2_ receptor activity make a detailed analysis of correlations difficult. We just note that the agonist activity decreases rapidly with increasing absolute value of the ΔcSAR(X), defined by Equation (2). It is illustrated in Figure 10. The ΔcSAR(CH_3_) parameters are close to one another for the C5 and C2 substitutions, like the respective differences in the HOMA indices. However, the C2-CH_3_ tautomer is much less active towards H_2_ receptors. It cannot be explained also by differences in the cSAR(aliph) parameters, substantially bigger for the C5-CH_3_ tautomer than for the C2-CH_3_ one.

**Scheme 2:**
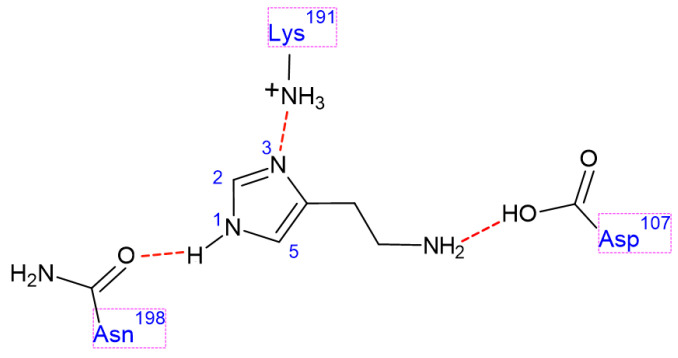
The possible molecular interactions of the optimized histamine molecule with the amino acids in the H_1_ receptor structure [35,36].

A molecule of histamine interacts with the amino acids of the receptor in various ways [2,33]. An explanatory drawing (Scheme 2) illustrates how the unsubstituted tautomer 1H interacts with the amino acids of the H_1_ receptor. The latter and the H_2_ receptors are sensitive to the side-chain protonated histamine tautomers [34]. This part of the study deals with the effect of substituents on the interactions with the H_1_ receptor.

The calculations commenced with the geometrically optimized 3H tautomer, subjected to the interactions with three amino acids of the H_1_ receptor. The geometry of the 3H-H_1_ complex was then optimized again, which resulted in isomerization into the 1H-H_1_. The final structure resembled that in Scheme 2. Thus, interactions with the H_1_ receptor shifted the isomerization equilibrium (Scheme 1) towards the 1H tautomer.

Substituents affect the molecular interactions of histamine with receptors. This causes changes in the intermolecular distances and disruption of the electron density. We considered four substituents: electron-withdrawing NO_2_, slightly π-donating but withdrawing by induction Cl, strongly π-donating NH_2_, and *σ*-donating by induction CH_3_. The results are reported in Table 3.

The distances between atoms in the N1─H⋯O bridge evidence that substituents change the proton-donating ability of the N1–H group rather weakly. Nevertheless, the strong electron-withdrawing NO_2_ substituent causes an elongation of the covalent bond N1─H and a shortening of the H⋯O distance, independently of the substituent position in the ring. The effect of substitution with the electron-donating NH_2_ group is the opposite. The values of *ρ* and positive ∇^2^*ρ* for the critical point of bond (BCP) and bond energies reported in Table S7 confirm the hydrogen bonding formation and follow those trends.

The electron-withdrawing substituents at the C2 and C5 atoms in the ring spectacularly influence the hydrogen bonding with the lysine residue in the H_1_ histamine receptor. N3 in the unsubstituted molecule of histamine strongly attracts the hydrogen atom of Lys-NH_3_^+^. Consequently, the equilibrium of the following reaction:

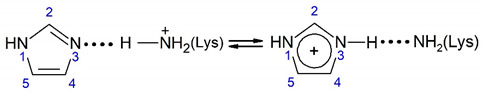

is shifted right, towards the imidazolium derivative. The electron-donating substituents at C2 reinforce the N3–H bond that is manifested in its shortening and elongation of the H⋯NH_2_(Lys) distance. On substitution with the electron-withdrawing groups, NO_2_ and Cl, the reaction equilibrium shifts left. The distance between N3 and H–NH_2_^+^(Lys) increases by about 50%. Similar effects occur on substitution at the C5 atom, except for the CH_3_ group. Surprisingly, this weak electron donor causes the reaction shifts left, towards the imidazole derivative. This cannot be explained at the present stage of the study. For the two tautomers in the equilibrium, CH_3_ substituted for H at C5 increases the negative charge at N3 and decreases the aromaticity expressed as the HOMA index. Rather, an opposite effect would be expected. A practical conclusion is obvious: knowledge about an isolated molecule can be insufficient for a correct prediction of its behavior when subjected to molecular interactions.

The aspartic acid residue (Asp)COO– in the H_1_ receptor attracts the hydrogen atom of the ^α^NH_3_^+^ strongly enough to form the (Asp)COO–H⋯^α^NH_2_ bond. In other words, it is a donor of a proton in hydrogen bonding. The bond length is just weakly sensitive to the substituent properties. Only the strongest electron acceptor, NO_2_, substituted at the C2 atom causes a significant elongation of the O–H bond and shortening of the H⋯^α^NH_2_ distance between the two molecules.

Indeed, molecular interactions involving the N3 atom and the terminal ^α^NH_2_ group of the histamine derivatives bond to the H_1_ receptor are more sensitive to the electron donor-acceptor properties of substituents than could be predicted solely from the cSAR(X) parameters for a given tautomer. The imidazole–imidazolium tautomerization that accompanies substitution enhances the difference between the cSAR(X) parameters. A comparison of the parameters for NO_2_, the strongest acceptor of electrons, and for NH_2_, the strongest donor, supports this idea. The cSAR(NO_2_) parameter is bigger for the imidazole derivatives than for the imidazolium ones, while the relation is opposite for cSAR(NH_2_). This is illustrated in Figure 2 for the C2 and C5 substitutions. Such an enhancement could not be predicted from the analysis of Hammett’s constants *σ*, because they characterize the substituents only, neglecting the electron structure of the ring. In our opinion, this is a cogent argument for the advantage of the cSAR-based approach supplemented by the analysis of the molecular interactions over that taking into account just the Hammett’s constants.

### 2.6. Stability of Substituted Histamine Systems

**Figure 11:**
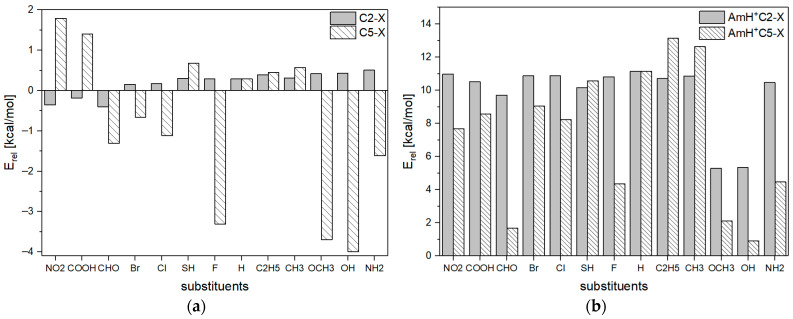
Differences between the total energies of 3H and 1H histamine derivatives, *E*_rel_ = *E*(3H) − *E*(1H), with various substituents at C2 and C5 atoms of the ring: neutral molecules (**a**) and cations with protonated NH_3_^+^ terminal group (**b**).

The neutral 1H-tautomer of histamine is more stable than the respective 3H one by about 0.3 kcal/mol, which is consistent with the experimental data [15]. Protonation of the terminal NH_2_ group enhances the difference in stabilization energies to 11.1 kcal/mol. The difference results from the N3 lone pair—π coupling, which promotes resonance (see Section 2.2.1). Substituents influence the stability of histamine tautomers, as is illustrated in Figure 11. The calculated relative energies, defined as differences between the total energies of the two compared tautomers, are reported in Table S8.

The relative energies *E*_rel_ for the C2 neutral tautomers are small. In general, the *E*_rel_(neutral C2) values follow the trend in the electron density, i.e., in the cSAR(X) parameters. Commonly, the 1H tautomers are more stable in this group. Only the strong acceptors of electrons, NO_2_, COOH, and CHO, substituted at the C2 atom, make the 3H tautomer more stable than the 1H one, and *E*_rel_(neutral C2) negative. This is probably due to the high aromaticity of the three derivatives, manifested in the high HOMA values (Table S5).

For the C5 substitution, the differences between the 3H and 1H tautomer are bigger. However, *E*_rel_(neutral C5) values are not correlated with the respective cSAR(X) parameters. This suggests stability of the C5-substituted systems disrupted by the substituents in the proximity of the sidechain. For this reason, an explanation of the *E*_rel_(neutral C5) trend solely in terms of the electron density fails.

Solvent water affects the stability of 1H(neutral) and 3H(neutral) substituted tautomers rather weakly. Significant differences in the stability in the vacuum and aqueous phase show just three derivatives with the NO_2_, COOH, and SH substituents at the C5 atom, cf. Table S8.

All 1H tautomers with the protonated nitrogen atom in the side chain are more stable than their 3H counterparts. As a rule, the *E*_rel_(AmH^+^) values are substantially bigger than the *E*_rel_(neutr), which is illustrated in Figure 11. Substituents influence the values of *E*_rel_(AmH^+^) randomly. We may just speculate that the hydrogen bond between the NH_3_^+^ terminal group of the chain and the oxygen atom of NO_2_, COOH, CHO, OCH_3,_ or OH substituted at C2 lowers the *E*_rel_(AmH^+^) value by ca. 5 kcal/mol. The latter estimation is based on the difference between *E*_rel_(AmH^+^) ≈ 5 kcal/mol for the OCH_3_ and OH substituted derivatives and *E*_rel_(AmH^+^) ≈ 10 kcal/mol for the remaining non-hydrogen-bonded ones (see Table S8). Imidazolium tautomers are more stable than respective ions with a protonated nitrogen atom of the side chain, which leads to negative values of *E*_rel_(ImH^+^) reported in Table S8.

## 3. Materials and Methods

### 3.1. Quantum Chemical Calculations

Mono-substituted histamine (4(5)-2′-aminoethylimidazole) tautomers in two neutral and three protonated forms and two conformations were studied (see Scheme 1). Moreover, interactions between the molecules of histamine and amino acids of the H_1_ receptor were simulated as well. Geometry and energy of tautomers were optimized with no symmetry constraints, using the DFT method with the B3LYP functional [37] implemented in the Gaussian16 package (Revision C.01) [38]. An aug-cc-pvtz basis set was applied to all involved atoms, and the empirical Grimme’s D3 dispersion correction was included in the calculation results. The SCF convergence criteria were adjusted to 10^−6^ hartree for both energy and density. Molecular structures represent the lowest energy minima on the potential energy surface. The obtained total energy is the sum of the electron energy and the nuclear repulsion energy, related to a reference state of infinitely separated nuclei and electrons.

The B3LYP/6-31g method was used to simulate structures of the histamine-receptor complexes. First, the molecule of histamine or its derivative was placed in the vicinity of three amino acid molecules arranged similarly as in the H1 receptor, cf. Scheme 2 and Refs. [35,36]. As the protonated form prevails in the physiological conditions, the AmH-3H^+^ tautomer was chosen. It proved to be not essential, because it isomerized into the AmH-1H^+^ in the optimization procedure.

To analyze the influence of substituents on the position of the proton involved in hydrogen bonding, the calculations were performed in which the donor-acceptor distances at three different reaction sites were frozen based on the optimized geometry of the unsubstituted system.

The substituent effect (SE) on the electron structure was analyzed by comparing the charge of the substituent active region (cSAR) parameters for the molecules of histamine and its derivatives [21,39]. The cSAR(X) values for substituents were calculated from the following Equation (3):

(3) $$cSAR\left(X\right)=q\left(X\right)+q{(C}_{ipso})$$

where X denotes a substituent in the imidazole (or imidazolium) ring, X = H for histamine, and *q* are the charges at the substituent X and the adjacent carbon atom in the ring. cSAR(X) > cSAR(H) for the electron-donating substituents, while the relation is opposite for the electron-accepting ones. Hirshfeld’s method of atomic charge assessment [40] was used in the calculation of cSAR values.

Changes in electron density are not confined to the nearest neighborhood of the substituted carbon atom but rather involve the entire molecule [21,39]. Thus, the cSAR(aliph) parameters, defined according to Equation (4):

(4) $$cSAR(aliph)=q(CH_{2}CH_{2}NH_{2})+q{(C}_{ipso}),$$

were calculated for the side chain of the molecule with the terminal ^α^NH_2_ group. For a comparison, the cSAR(NH_2_) parameters were also calculated:

(5) $$cSAR(NH_{2})=q(NH_{2})+q{(C}_{ipso}),$$

To assess the influence of a solvent on the substituent effect, we repeated the calculations for the molecules in a solvent modelled as a continuum of uniform electric permittivity rather than in a vacuum. The Polarizable Continuum Model (PCM) model [41] was applied to the 1H(neutral) tautomers. The ΔcSAR(X) and ΔcSAR(aliph) values (Equation (2)) obtained in vacuum and in solvent water are highly correlated (see Figure S3) and show no statistically significant differences, *p* > 0.52 as per the result of *t*-testing. Thus, although the solvent with high electric permittivity changes the cSAR values, it does not disrupt the trends. For other solvents with a smaller electric permittivity, the difference would be smaller.

Since substituents affect the electron distribution in the ring, we also calculated the geometry-based Harmonic Oscillator Model of Aromaticity (HOMA) indices. The HOMA index, which describes the π-electron delocalization in the imidazole ring, is defined by Equation (6) [42]:

(6) $$HOMA=1-\sum_{i=1}^{2}\frac{\alpha_{i}}{n}\sum_{j=1}^{n}{\left(d_{opt,i}-d_{i,j}\right)}^{2}$$

where *i* denotes the type of bond, *i* = 1 for the CC and *i* = 2 for the CN, and *n* is the number of bonds of the given type. The coefficient *α_i_* is an empirical normalization constant for the bond of *i*-type, *d*_opt,*i*_ is the bond length in an “ideal aromatic” system, while *d_i,j_* is the actual length of bond *j* in the real molecule.

To describe the nature of bonding and electronic interactions within the system, bond critical points (BCPs) at the locations corresponding to zero gradient of the electron density were identified. Topological parameters: the electron density *ρ*, its Laplacian ∇^2^*ρ*, and potential energy density, *V*(*r*), for BCPs between pairs of atoms were obtained within the framework of Bader’s Atoms in Molecules (AIM) theory [43]. To this aim, the Multiwfn program v. 3.7 [44] was applied, with the wave functions obtained from the DFT. The energy of molecular interactions was calculated from the following Equation (7) of Afonin et al. [45]:

(7) $$E_{HB}=191.4\cdot \rho -1.78$$

where *ρ* is the electron density (in e/bohr^−3^) at the BCP. This equation can be used to various types of non-covalent interactions, i.a., OH⋯O, OH⋯N, OH⋯halogen, NH⋯O, NH⋯N, CH⋯O, CH⋯N and CH⋯halogen. In this case, positive values of *E*_HB_ are interpreted as “bond strength”; the higher the value, the more stable the bond [45].

### 3.2. Regression Analysis

Model fittings and correlation analyses were performed with the Statistica v. 13 software [46]. The basic model was that of simple regression:

(8) y=a·x+b,

with two fitted coefficients: the slope *a* and the intercept coefficient *b*. In each series of fittings, variable *x* remained the same, and *y* changed. For example, in the correlations of Hammett’s constants σ with the cSAR(X) parameters, *σ* was the independent variable and cSAR(X) the dependent one. That was because several cSAR(X) vectors corresponded to one *σ* vector.

The quality of the fit was characterized by the values of the coefficient of determination *R*^2^, defined as:

(9) $$R^{2}=1-\frac{\sum {\left(y_{i}-{\hat{y}}_{i}\right)}^{2}}{\sum {\left(y_{i}-\bar{y}\right)}^{2}},$$

where *y_i_* is the data value associated with a value ${\hat{y}}_{i}$ predicted from the model, and $\bar{y}$ is the arithmetic mean of *y_i_*. Note that the coefficients of determination for the model equations with and without the adjustable intercept coefficients cannot be compared directly. Following Anscombe’s recommendations [47], we also analysed the graphs of the fitted functions and residuals, as well as the distributions of the residuals. The statistical significance of the coefficients in the regression Equation (8) was *t*-tested by applying the procedures implemented in the Statistica software [46].

## 4. Conclusions

The approach based on the charge of the substituent active region (cSAR) proved to be an efficient tool for the analysis of the substituent effect (SE) in the molecules of histamine derivatives. In particular, the cSAR(X) parameters characterize the electron density in the X-substituted ring better than the conventional Hammett’s constants *σ*. Values of the latter for para-substituents agree well with the cSAR(X) predictions for the substituents at the C2 atom in the imidazole ring, for both neutral and cationic tautomers. For the imidazolium tautomer, with the positive charge spread over the ring, the agreement is worse. A qualitative agreement is preserved, i.e., the strong electron donors, such as NH_2_, and the strong acceptors, e.g., NO_2_, are classified as such substituents by both methods. However, the substituents which change the electron density in the ring weakly can be classified incorrectly if based on Hammett’s constants *σ*_p_. For example, SH, F, Cl, and Br are weak electron donors to the imidazolium ring, as evidenced by the differences between the cSAR(X) and cSAR(H) parameters, while the *σ*_p_ values suggest the opposite.

For the substituents at the C5 atom, the agreement is worse. Indeed, Hammett’s constants correctly describe changes in the ring charge only for the substitution in the neutral tautomers. This study also evidenced that predictions based on *σ*_m_ indices, those for *meta* substitution, would give worse results than those based on *σ*_p_.

The cSAR approach applied to the side chain of histamine tautomers evidenced that the charge density on the terminal NH_2_ group of the chain is correlated with the electron-withdrawing ability of the substituents. Thus, the stronger the electron acceptor substituted in the ring, the lower the cSAR(NH_2_) parameter. This also holds for the imidazolium tautomers. The regularity was not confirmed for protonated tautomers with the NH_3_^+^ terminal group and substituents at the C5 atom of the ring. This counterintuitive observation evidences that the terminal group is screened by the hydrocarbon chain. Indeed, the cSAR(aliph) parameters, in which the whole side chain is considered, are correlated with the cSAR(X) ones. Intramolecular hydrogen bonds between the NH_3_^+^ group and substituents, e.g., NO_2_, CHO, and COOH, at the *ortho* C5 atom of the ring reduce the charge on the chain. Thus, the correlation analysis could reveal the intramolecular interactions. The intramolecular hydrogen bonds probably account for the lack of correlation between the cSAR parameters and the stability of the ^α^NH_2_ protonated tautomers.

Aromaticity of the histamine molecules substituted at the C2 atom is related to the cSAR(X) parameters. As a measure of the former, we applied the indices based on the Harmonic Oscillator Model of Aromaticity (HOMA). At least a half-quantitative relationship occurs for all five C2 tautomers. In particular, the HOMA indices are almost perfectly correlated with the cSAR(X) parameters for the C2-ImH^+^ tautomer. This is probably due to its symmetry: the substituent is located in the ring between two NH groups. For less symmetrical tautomers, the correlation is worse; nevertheless, the cSAR(X) parameters still facilitate the assessment of aromaticity.

A similar attempt at correlating the cSAR(X) parameters with the HOMA indices failed for the C5-substituted tautomers. Only the results for 1H(neutral) and AmH-1H^+^ tautomers were acceptable. The reason is probably the lower symmetry of the molecules. However, a deeper interpretation would require further studies of similar systems. At the present stage, we can just conclude that substituents at the C2 atom affect the aromaticity more strongly than those at the C5 position, which confirms the recent suggestion by Wieczorkiewicz et al. [30].

Last but not least, the cSAR approach showed a clear advantage over that based on Hammett’s constants *σ*. Indeed, a theoretical analysis of molecular interactions between histamine derivatives and their receptors requires knowledge of whether they are imidazole or imidazolium tautomers. Information about the electron structure gathered from studies of isolated molecules may be insufficient. Histamine tautomers may isomerise on hydrogen bonding with the receptors. This study suggested that the 3H tautomer isomerises into the 1H one after binding to the H_1_ receptor. Moreover, the electron structure of the histamine derivative hydrogen-bonded to the receptor may strongly depend on the electron donor-acceptor properties of the substituent. The results of our calculations suggested that the strong electron-accepting substituents, e.g., NO_2_, favor the imidazole configuration of the ring in the bonded molecule, while the strong electron-donating ones, e.g., NH_2_, promote the imidazolium one.

Understanding interactions of histamine derivatives with the receptors is crucial for the development of antihistamine drugs for diverse therapeutic applications. Analysis of atomic charges facilitates the identification of active sites in the molecules. Thus, basic research may help in creating a library of antihistamine-active compounds. However, the results of this study, although promising, should be considered as preliminary.

## Figures and Tables

**Table 1:** Coefficients *a* and *b* of the regression Equation (8) with standard errors and respective coefficients of determination *R*^2^ for the correlations of the Hammett’s constant *σ* with ΔcSAR(X) parameters.

Substituent Position	Hammett’s Constant	Tautomer	*a*	*S_a_*	*B*	*S_b_*	*R* ^2^
2	*σ*_p_	1H, 3H, AmH-1H^+^,	0.179	0.009			0.885
		AmH-3H^+^					
		IMH^+^	0.192	0.018	–0.056	0.007	0.922
2	*σ*_m_	1H, 3H	0.290	0.039	–0.047	0.013	0.738
		AmH-1H^+^, AmH-3H^+^	0.273	0.032	–0.069	0.011	0.783
		IMH^+^	0.296	0.055	–0.115	0.019	0.764
5	*σ*_p_	1H, 3H	0.172	0.012			0.888
		AmH-1H^+^, AmH-3H^+^	0.125	0.011	–0.020	0.004	0.845
		IMH^+^	0.116	0.034	–0.054	0.013	0.533
5	*σ*_m_	1H, 3H	0.259	0.035	–0.043	0.012	0.727
		AmH-1H^+^, AmH-3H^+^	0.198	0.027	–0.060	0.009	0.732
		IMH^+^	0.204	0.061	–0.096	0.021	0.556

**Table 2:** H_2_ receptor activities (RA) [32] and theoretical characteristics of studied 1H(neutral) histamine derivatives.

	C5-X					C2-X	
X	H	CH3	Cl	Br	NO2	H	CH3
RA	100	43	11	9	0.6	100	4
cSAR(X)	0.0016	0.0413	−0.0187	−0.0165	−0.1616	0.0881	0.1195
ΔcSAR(X)	0	−0.0397	0.0203	0.0181	0.1632	0	−0.0314
cSAR(aliph)	0.0310	0.0065	0.0275	0.0255	0.0963	0.0314	0.0215
ΔcSAR(aliph)	0	0.0245	0.0035	0.0055	−0.0653	0	0.0099
HOMA	0.8790	0.8667	0.8766	0.8785	0.9397	0.8790	0.8644

**Table 3:** Non-covalent bonds parameters: distances *d* (in Å) and the charge density *ρ* (in e/bohr^3^) and its Laplacian ∇^2^*ρ* (in e/bohr^5^) calculated for the bond critical points between atom pairs of histamine and the amino acid of the receptor H_1_.

X	N1–H⋯O				N3⋯H–N				H_2_^α^N⋯H–O			
	*d*_N–H_	*d*_H__⋯O_	*Ρ*	∇^2^*ρ*	*d*_N–H_	*d*_H__⋯N_	*ρ*	∇^2^*ρ*	*d*_N__⋯H_	*d*_O–H_	*ρ*	∇^2^*ρ*
C2-NO_2_	1.06	1.56	0.062	0.202	1.73	1.06	0.044	0.127	1.33	1.18	0.084	0.138
C2-Cl	1.03	1.67	0.043	0.173	1.63	1.09	0.057	0.146	1.47	1.09	0.097	0.109
H	1.03	1.70	0.042	0.163	1.14	1.50	0.070	0.154	1.49	1.08	0.099	0.101
C2-CH_3_	1.04	1.69	0.043	0.163	1.08	1.67	0.063	0.137	1.63	1.04	0.068	0.150
C2-NH_2_	1.01	1.91	0.042	0.163	1.09	1.62	0.070	0.154	1.49	1.09	0.083	0.142
C5-NO_2_	1.07	1.56	0.065	0.207	1.69	1.08	0.057	0.148	1.44	1.11	0.104	0.837
C5-Cl	1.04	1.63	0.052	0.186	1.64	1.09	0.064	0.153	1.46	1.10	0.100	0.101
C5-CH_3_	1.03	1.75	0.036	0.143	1.59	1.11	0.071	0.153	1.47	1.09	0.097	0.109
C5-NH_2_	1.03	1.80	0.035	0.129	1.09	1.64	0.064	0.147	1.65	1.04	0.064	0.147

## Data Availability

The original contributions presented in this study are included in the article. Further inquiries can be directed to the first author (A.J.).

## Supplementary Materials

The following supporting information can be downloaded at: https://www.mdpi.com/article/10.3390/molecules30183736/s1, Table S1: The obtained values of cSAR parameters for neutral and monocationic forms of C2-X (a) and C5-X (b) histamine derivatives in *trans* conformation; Table S2: Parameters of the regression Equation (8) for the correlations of the cSAR(X) with (a) cSAR(aliph) and (b) cSAR(NH_2_): the slope coefficients *a* with standard errors *s_a_* and ranges from *a*_min_ to *a*_max_ at the 95% level of confidence, determination coefficients *R*^2^, and numbers of data pairs in the fitting procedure N; Table S3: The charges at the N and NH atoms in C2 and C5-substituted histamine derivatives in their neutral (a) and cationic forms (b); Table S4: Regression coefficients of the electron density at protonated N atom vs. cSAR(X) Equation (8), *q* = *a*·cSAR(X) + *b*, with their standard errors *s* and coefficients of determination *R*^2^; Table S5: The HOMA indices for C2 (a) and C5 (b) studied histamines in neutral and cationic forms; Table S6: The slope coefficients of the HOMA vs. cSAR(X) Equation (8), HOMA = *a*·cSAR(X) + *b*, with their standard errors *s_a_*, ranges from *a*_min_ to *a*_max_ at the confidence level of 95%, and coefficients of determination *R*^2^; Table S7: Estimated energies of hydrogen bonds *E*_HB_ (in kcal/mol) at bond critical points (BCPs) between the histamine derivatives and the amino acid residues of the receptor H_1_; Table S8. The relative energies *E*_rel_ (in kcal/mol) for the histamine molecules and their C2 and C5 substituted derivatives in neutral and cationic forms in vacuum and water (PCM); Table S9: The values of cSAR parameters for neutral C2-X and C5-X histamine 1H tautomer derivatives obtained in a solvent water environment using the PCM method. Figure S1: Simple regression lines (Equation (8)) fitted to the cSAR(aliph) vs. cSAR(X) sets of data for all histamine systems substituted at the C2 and C5 atoms in neutral (a), and cationic forms (b); Figure S2: Simple regression lines (Equation (8)) fitted to the cSAR(NH_2_) vs. cSAR(X) sets of data for all histamine systems substituted at the C2 and C5 atoms in neutral (a) and AmH^+^ cationic (b) forms. Figure S3: Simple regression lines (Equation (8) with the intercept coefficient *b* = 0) fitted to the ΔcSAR(X)_PCM_ vs. ΔcSAR(X)_GP_ (a) and ΔcSAR(aliph)_PCM_ vs. ΔcSAR(aliph)_GP_ (b) sets of data for all histamine systems substituted at the C2 and C5 atoms in neutral 1H forms; the subscripts PCM and GP denote solvent water and vacuum, respectively.
