# Supplementary material for: Substituent Effect in Histamine and Its Impact on Interactions with the G Protein-Coupled Human Receptor H1 Modelled by Quantum-Chemical Methods
Source: Molecules. 2025 Sep 15;30(18):3736. doi: 10.3390/molecules30183736 (PMC12472401; doi:10.3390/molecules30183736)
Supplement: Supplementary file 1 [file molecules-30-03736-s001.zip › molecules-3784226-supplementary.pdf]

Supplementary Materials

Substituent Effect in Histamine and Its Impact on Interactions with the G Protein-Coupled Human Receptor H_1 Modelled by Quantum-Chemical Methods

Anna Jezuita [1], Małgorzata Makowska-Janusik [1], Krzysztof Ejsmont [2] and Wojciech Marczak [1,*]

[1] Faculty of Science and Technology, Jan Dlugosz University, Al. Armii Krajowej 13/15, 42-200 Czestochowa, Poland; a.jezuita@ujd.edu.pl (A.J.); m.makowska@ujd.edu.pl (M.M.-J.)

[2] Department of Chemistry and Pharmacy, University of Opole, Oleska 48, 45-052 Opole, Poland;
eismont@uni.opole.pl

* Correspondence: w.marczak@ujd.edu.pl

The calculation results not included in these Supplementary Materials can be obtained for personal use from the first author (A.J.)

Table S1. The values of cSAR parameters for neutral and monocationic C2-X (a) and C5-X (b) histamine derivatives in *trans* conformation.

(a) C2-X	σ_p	σ_m	3H (neutral)			1H (neutral)			AmH-3H$^+$			AmH-1H$^+$			ImH$^+$		
			cSAR (X)	cSAR ($^{\alpha}NH_2$)	cSAR (aliph)	cSAR (X)	cSAR ($^{\alpha}NH_2$)	cSAR (aliph)	cSAR (X)	cSAR ($^{\alpha}NH_2$)	cSAR (aliph)	cSAR (X)	cSAR ($^{\alpha}NH_2$)	cSAR (aliph)	cSAR (X)	cSAR ($^{\alpha}NH_2$)	cSAR (aliph)
NO_2	0.78	0.71	-0.0910	-0.0144	0.1204	-0.0816	-0.0248	0.0860	0.0128	0.7263	0.9745	0.0024	0.7059	0.9295	0.1469	0.0412	0.2817
COOH	0.45	0.37	-0.0346	-0.0211	0.0956	-0.0258	-0.0315	0.0592	0.0682	0.7196	0.9504	0.0589	0.6981	0.9012	0.2092	0.0336	0.2537
CHO	0.42	0.35	-0.0495	-0.0193	0.1058	-0.0393	-0.0298	0.0625	0.0566	0.7212	0.9589	0.0494	0.7001	0.9049	0.1936	0.0360	0.2590
Br	0.23	0.39	0.0466	-0.0239	0.0675	0.0525	-0.0337	0.0497	0.1313	0.7175	0.9281	0.1269	0.6966	0.8927	0.2869	0.0306	0.2345
Cl	0.23	0.37	0.0480	-0.0240	0.0659	0.0537	-0.0338	0.0491	0.1271	0.7179	0.9283	0.1228	0.6970	0.8938	0.2734	0.0315	0.2371
SH	0.15	0.25	0.0856	-0.0269	0.0525	0.0916	-0.0366	0.0370	0.1730	0.7141	0.9111	0.1670	0.6934	0.8799	0.2823	0.0299	0.2349
F	0.06	0.34	0.0721	-0.0248	0.0571	0.0770	-0.0343	0.0472	0.1332	0.7184	0.9249	0.1303	0.6981	0.8964	0.2557	0.0333	0.2400
H	0.00	0.00	0.0830	-0.0281	0.0509	0.0881	-0.0382	0.0314	0.1413	0.7150	0.9188	0.1373	0.6934	0.8804	0.2440	0.0310	0.2374
C_2H_5	-0.15		0.1044	-0.0305	0.0399	0.1134	-0.0403	0.0227	0.1829	0.7111	0.9016	0.1787	0.6894	0.8652	0.3446	0.0248	0.2104
CH_3	-0.17	-0.07	0.1137	-0.0303	0.0406	0.1195	-0.0402	0.0215	0.1865	0.7118	0.9037	0.1827	0.6900	0.8663	0.3219	0.0264	0.2183
OCH_3	-0.27	0.12	0.1315	-0.0305	0.0352	0.1371	-0.0398	0.0224	0.2150	0.7109	0.8952	0.2144	0.7035	0.8907	0.3865	0.0247	0.2019
OH	-0.37	0.12	0.1331	-0.0292	0.0384	0.1381	-0.0384	0.0285	0.2041	0.7131	0.9021	0.2050	0.7057	0.8996	0.3446	0.0279	0.2145
NH_2	-0.66	-0.16	0.1569	-0.0315	0.0297	0.1647	-0.0409	0.0197	0.2475	0.7093	0.8846	0.2464	0.6885	0.8610	0.4176	0.0228	0.1976
Δ	1.44	0.87	0.2479	0.0170	0.0906	0.2463	0.0161	0.0663	0.2347	0.0169	0.0899	0.2440	0.0174	0.0685	0.2707	0.0184	0.0841
average	0.05	0.23	0.0615	-0.0257	0.0615	0.0684	-0.0356	0.0413	0.1446	0.7159	0.9217	0.1402	0.6969	0.8894	0.2852	0.0303	0.2324
SD	0.38	0.24	0.0764	0.0051	0.0289	0.0752	0.0048	0.0200	0.0675	0.0049	0.0265	0.0704	0.0059	0.0189	0.0776	0.0051	0.0239

(b) C5-X	σ_p	σ_m	3H (neutral)			1H (neutral)			AmH-3H$^+$			AmH-1H$^+$			ImH$^+$		
			cSAR (X)	cSAR ($^{\alpha}NH_2$)	cSAR (aliph)	cSAR (X)	cSAR ($^{\alpha}NH_2$)	cSAR (aliph)	cSAR (X)	cSAR ($^{\alpha}NH_2$)	cSAR (aliph)	cSAR (X)	cSAR ($^{\alpha}NH_2$)	cSAR (aliph)	cSAR (X)	cSAR ($^{\alpha}NH_2$)	cSAR (aliph)
NO_2	0.78	0.71	-0.1554	-0.0195	0.1018	-0.1616	-0.0237	0.0963	-0.0762	0.6955	0.9293	-0.0576	0.5785	0.8605	0.0281	0.0447	0.2762
COOH	0.45	0.37	-0.1067	-0.0255	0.0879	-0.1031	-0.0321	0.0765	-0.0233	0.6913	0.9128	0.0311	0.5500	0.8126	0.0856	0.0343	0.2545
CHO	0.42	0.35	-0.1140	-0.0248	0.0995	-0.1174	-0.0306	0.0843	-0.0451	0.6970	0.9285	-0.0024	0.5431	0.8205	0.2280	0.0369	0.2625
Br	0.23	0.39	-0.0352	-0.0293	0.0442	-0.0165	-0.0380	0.0255	0.0284	0.6994	0.8792	0.0450	0.6976	0.8755	0.1520	0.0281	0.2093
Cl	0.23	0.37	-0.0371	-0.0288	0.0477	-0.0187	-0.0381	0.0275	0.0185	0.7023	0.8875	0.0378	0.6981	0.8800	0.1374	0.0295	0.2164
SH	0.15	0.25	-0.0250	-0.0305	0.0440	-0.0192	-0.0381	0.0299	0.0480	0.7061	0.8789	0.0488	0.6958	0.8770	0.1503	0.0288	0.2164
F	0.06	0.34	-0.0136	-0.0296	0.0353	0.0013	-0.0398	0.0170	0.0121	0.7107	0.8969	0.0442	0.6983	0.8770	0.1239	0.0298	0.2188
H	0.00	0.00	-0.0068	-0.0281	0.0509	0.0016	-0.038	0.031	0.0136	0.7150	0.9188	0.0469	0.6934	0.8804	0.1134	0.0310	0.2374
C_2H_5	-0.15		0.0153	-0.0349	0.0322	0.0400	-0.0448	0.0076	0.0530	0.7085	0.8855	0.1023	0.6883	0.8535	0.1948	0.0221	0.1970
CH_3	-0.17	-0.07	0.0237	-0.0350	0.0305	0.0413	-0.0454	0.0065	0.0563	0.7101	0.8909	0.0969	0.6891	0.8555	0.1727	0.0289	0.2137
OCH_3	-0.27	0.12	0.0347	-0.0374	0.0121	0.0467	-0.0443	-0.0004	0.0912	0.6980	0.8523	0.1448	0.6877	0.8375	0.2473	0.0223	0.1798
OH	-0.37	0.12	0.0382	-0.0356	0.0181	0.0610	-0.0434	-0.0164	0.0807	0.7028	0.8660	0.0931	0.6932	0.8611	0.1714	0.0459	0.2065
NH_2	-0.66	-0.16	0.0603	-0.0381	0.0029	0.0616	-0.0450	-0.0084	0.0930	0.6909	0.862	0.1079	0.6904	0.8568	0.2379	0.0205	0.1681
Δ	1.44	0.87	0.2158	0.0186	0.0988	0.2232	0.0217	0.1127	0.1692	0.0241	0.0770	0.2024	0.1552	0.0678	0.2192	0.0254	0.1081
average	0.05	0.23	-0.0247	-0.0306	0.0467	-0.0141	-0.0386	0.0290	0.0269	0.7021	0.8914	0.0568	0.6618	0.8575	0.1571	0.0310	0.2197
SD	0.38	0.24	0.0653	0.0055	0.0317	0.0719	0.0065	0.0357	0.0518	0.0076	0.0249	0.0528	0.0602	0.0222	0.0624	0.0079	0.0312

Table S2 Parameters of the regression Equation 8 for the correlations of the cSAR(X) with (a) cSAR(aliph) and (b) cSAR(NH_2): the slope coefficients *a* with standard errors s_a and ranges from a_{min} to a_{max} at the 95% level of confidence, determination coefficients R^2, and numbers of data pairs in the fitting procedure *N*

**(a)**			cSAR(aliph) vs cSAR(X)				
**Tautomer**	**X**	***N***	***a***	s_a	a_{min}	a_{max}	R^2
3H	2	13	-0.376	0.010	-0.399	-0.353	0.992
1H		13	-0.254	0.022	-0.304	-0.205	0.921
AmH-3H$^+$		13	-0.390	0.012	-0.416	-0.364	0.990
AmH-1H$^+$		13	-0.210	0.050	-0.321	-0.100	0.614
ImH$^+$		13	-0.304	0.015	-0.337	-0.271	0.974
3H	5	13	-0.475	0.032	-0.545	-0.405	0.953
1H		13	-0.491	0.022	-0.540	-0.443	0.978
AmH-3H$^+$		13	-0.445	0.055	-0.566	-0.325	0.858
AmH-1H$^+$		3[a]	-0.558	0.130	-2.211	1.094	0.949
AmH-1H$^+$		10[b]	-0.392	0.022	-0.442	-0.343	0.976
AmH-1H$^+$ (co-planar)		13	-0.431	0.030	-0.504	-0.364	0.960
ImH$^+$		13	-0.347	0.108	-0.585	-0.109	0.484
ImH$^+$		12[c]	-0.472	0.033	-0.546	-0.399	0.954

[a] Correlation for three substituents in AmH-1H$^+$: NO_2, COOH, CHO

[b] Correlation for the remaining 10 substituents in AmH-1H$^+$

[c] Correlation for 12 substituents, the outlying data pair for CHO was rejected

**(b)**			cSAR(NH_2) vs cSAR(X)				
**Tautomer**	**X**	***N***	***a***	s_a	a_{min}	a_{max}	R^2
3H	2	13	-0.065	0.005	-0.135	0.004	0.945
1H		13	-0.062	0.005	-0.132	0.008	0.923
AmH-3H$^+$		13	-0.070	0.005	-0.140	0.000	0.936
AmH-1H$^+$		13	-0.036	0.023	-0.106	0.034	0.185
ImH$^+$		13	-0.063	0.005	-0.134	0.000	0.932
3H	5	13	-0.080	0.007	-0.150	-0.010	0.916
1H		13	-0.089	0.005	-0.159	-0.019	0.961
AmH-3H$^+$		13	-0.025	0.044	-0.045	0.094	0.029
AmH-1H$^+$		13	0. 732	0.264	-0.661	0.802	0.411
AmH-1H$^+$		10[b]	-0.102	0.017	-0.172	-0.032	0.820
ImH$^+$		13	-0.070	0.032	-0.140	0.000	0.309
ImH$^+$		12[c]	-0.090	0.030	-0.160	-0.020	0.476

[b] Correlation for 10 substituents in AmH-1H$^+$ (NO_2, COOH, and CHO excluded)

[c] Correlation for 12 substituents, the outlying data pair for CHO was rejected

Table S3. The charges at the N and NH atoms in C2 and C5-substituted histamine derivatives in their neutral (a) and cationic forms (b).

	C2 substitution				C5 substitution			
(a)	3H (neutral)		1H (neutral)		3H (neutral)		1H (neutral)	
	qN3H	qN1	qN1H	qN3	qN3H	qN1	qN1H	qN3
NO_2	0.1112	-0.1708	0.1146	-0.1702	0.1143	-0.1799	0.1123	-0.1873
COOH	0.1092	-0.1831	0.1121	-0.1812	0.1043	-0.1882	0.1031	-0.1967
CHO	0.1137	-0.1847	0.1167	-0.1832	0.1064	-0.1928	0.1049	-0.1935
Br	0.0865	-0.2055	0.0874	-0.2013	0.0966	-0.2027	0.0894	-0.1964
Cl	0.0884	-0.2067	0.0893	-0.2023	0.0959	-0.2033	0.0914	-0.1970
SH	0.0826	-0.2177	0.0828	-0.2124	0.0946	-0.2070	0.0865	-0.1983
F	0.0865	-0.2184	0.0867	-0.2133	0.0925	-0.2106	0.0905	-0.1975
H	0.0861	-0.2132	0.0870	-0.2081	0.0861	-0.2132	0.0870	-0.2081
C_2H_5	0.0763	-0.2061	0.0778	-0.2058	0.0794	-0.2072	0.0823	-0.2100
CH_3	0.0774	-0.2180	0.0775	-0.2123	0.0794	-0.2136	0.0811	-0.2093
OCH_3	0.0748	-0.2281	0.0738	-0.2218	0.0853	-0.2104	0.0824	-0.2070
OH	0.0806	-0.2428	0.0801	-0.2365	0.0858	-0.2262	0.0858	-0.2059
NH_2	0.0672	-0.2396	0.0652	-0.2335	0.0827	-0.2247	0.0787	-0.2102
Δ	0.0465	0.0721	0.0515	0.0663	0.0349	0.0463	0.0336	0.0229
average	0.0877	-0.2103	0.0885	-0.2063	0.0926	-0.2061	0.0904	-0.2013
SD	0.0147	0.0213	0.0162	0.0194	0.0109	0.0132	0.0102	0.0074

	C2 substitution						C5 substitution					
(b)	$AmH\text{-}3H^+$		$AmH\text{-}1H^+$		ImH^+		$AmH\text{-}3H^+$		$AmH\text{-}1H^+$		ImH^+	
	qN3H	qN1	qN1H	qN3	qN3H	qN1H	qN3H	qN1	qN1H	qN3	qN3H	qN1H
NO_2	0.1222	-0.1498	0.1510	-0.1681	0.2093	0.2186	0.1313	-0.1556	0.1543	-0.1618	0.2141	0.2083
COOH	0.1202	-0.1623	0.1478	-0.1784	0.1967	0.2116	0.1206	-0.1636	0.1431	-0.1711	0.2047	0.2003
CHO	0.1238	-0.1643	0.1514	-0.1817	0.1996	0.2123	0.1463	-0.1582	0.1516	-0.1638	0.2077	0.2051
Br	0.1010	-0.1820	0.1264	-0.1988	0.1808	0.1873	0.1154	-0.1769	0.1282	-0.1922	0.2020	0.1928
Cl	0.1042	-0.1818	0.1295	-0.1988	0.1857	0.1922	0.1158	-0.1763	0.1313	-0.1925	0.2034	0.1973
SH	0.0968	-0.1952	0.1211	-0.2104	0.1813	0.1898	0.1090	-0.1832	0.1262	-0.1963	0.1982	0.1906
F	0.1066	-0.1890	0.1311	-0.2070	0.1936	0.1995	0.1163	-0.1806	0.1332	-0.1931	0.2058	0.2037
H	0.1085	-0.1821	0.1336	-0.1981	0.1992	0.2061	0.1085	-0.1821	0.1336	-0.1981	0.1992	0.2061
C_2H_5	0.0937	-0.1821	0.1179	-0.1978	0.1683	0.1792	0.1005	-0.1717	0.1202	-0.2034	0.1904	0.1876
CH_3	0.0955	-0.1913	0.1196	-0.2060	0.1774	0.1839	0.1016	-0.1838	0.1217	-0.2025	0.1901	0.1889
OCH_3	0.0905	-0.2066	0.1082	-0.2168	0.1475	0.1708	0.1056	-0.1864	0.1021	-0.1997	0.1927	0.1661
OH	0.0982	-0.2162	0.1158	-0.2273	0.1604	0.1816	0.1077	-0.1986	0.1161	-0.2000	0.1948	0.1946
NH_2	0.0794	-0.2175	0.1018	-0.2333	0.1485	0.1522	0.1046	-0.1955	0.1097	-0.2030	0.1917	0.1795
Δ	0.0444	0.0677	0.0495	0.0652	0.0619	0.0665	0.0458	0.0430	0.0522	0.0415	0.0239	0.0423
average	0.1031	-0.1862	0.1273	-0.2017	0.1806	0.1911	0.1141	-0.1779	0.1286	-0.1906	0.1996	0.1939
SD	0.0131	0.0201	0.0157	0.0185	0.0198	0.0186	0.0129	0.0130	0.0153	0.0149	0.0075	0.0119

Table S4 Regression coefficients of the electron density at protonated N atom vs cSAR(X) Equation 8, $q = a \cdot \text{cSAR(X)} + b$, with their standard errors s and coefficients of determination R^2.

	Tautomer	X[a]	Protonated N[b]		Charge at N[c]	$-a$	s_a	b	s_b	R^2
			1N	3N						
1	3H	2	-	1	3	0.187	0.014	0.099	0.001	0.942
2	3H	2	-	1	1	0.264	0.026	-0.194	0.003	0.901
3	AmH-3H+	2	-	1	3	0.186	0.017	0.130	0.003	0.917
4	AmH-3H+	2	-	1	1	0.282	0.029	-0.145	0.005	0.895
5	1H	2	1	-	1	0.209	0.014	0.103	0.001	0.950
6	1H	2	1	-	3	0.246	0.023	-0.189	0.002	0.909
7	AmH-1H+	2	1	-	1	0.217	0.015	0.158	0.002	0.975
8	AmH-1H+	2	1	-	3	0.249	0.026	-0.167	0.004	0.893
9	ImH+	2	1	1	3	0.246	0.019	0.251	0.006	0.917
10	ImH+	2	1	1	1	0.234	0.015	0.258	0.004	0.956
11	3H	5	-	1	3	0.158	0.016	0.089	0.001	0.904
12	3H	5	-	1	1	0.192	0.019	-0.211	0.001	0.900
13	AmH-3H+	5	-	1	3	0.209	0.040	0.120	0.002	0.709
14	AmH-3H+	5	-	1	1	0.172	0.002	-0.227	0.033	0.815
15	1H	5	1	-	1	0.138	0.010	0.088	0.001	0.944
16	1H	5	1	-	3	0.091	0.015	-0.203	0.001	0.779
17	AmH-1H+	5	1	-	1	0.276	0.026	0.144	0.002	0.911
18	AmH-1H+	5	1	-	3	0.240	0.045	-0.177	0.003	0.722
19	ImH+	5	1	1	3	0.080	0.027	0.212	0.005	0.448
20	ImH+	5	1	1	1	0.137	0.040	0.215	0.007	0.518

[a]Number of the substituted carbon atom in the ring; [b]Location of the protonated nitrogen atom in the ring, 1 or/and 3; [c]Number of the nitrogen atom in the ring with the charge q correlated with cSAR(X)

Table S5. The HOMA indices for C2 (a) and C5 (b) substituted histamines in neutral and cationic forms.

(a)	HOMA				
**C2-X**	**3H (neutral)**	**1H (neutral)**	**AmH-3H⁺**	**AmH-1H⁺**	**ImH⁺**
NO_2	0.9306	0.9327	0.9191	0.9307	0.9137
COOH	0.9372	0.9414	0.9326	0.9409	0.9169
CHO	0.9444	0.9481	0.9381	0.9466	0.9241
Br	0.8441	0.8500	0.8682	0.8731	0.8410
Cl	0.8421	0.8483	0.8666	0.8711	0.8359
SH	0.8404	0.8475	0.8647	0.8655	0.8819
F	0.7917	0.7991	0.8299	0.8312	0.7749
H	0.8733	0.8790	0.8890	0.8944	0.8628
C_2H_5	0.8543	0.8701	0.8739	0.8784	0.8342
CH_3	0.8572	0.8644	0.8763	0.8790	0.8369
OCH_3	0.8024	0.8102	0.8421	0.8541	0.7498
OH	0.7993	0.8075	0.8409	0.8525	0.7502
NH_2	0.8146	0.8200	0.8400	0.8327	0.7229
Δ	0.1527	0.1490	0.1082	0.1154	0.2012
average	0.8563	0.8629	0.8755	0.8808	0.8342
SD	0.0523	0.0508	0.0356	0.0379	0.0674

(b)	HOMA				
**C5-X**	**3H (neutral)**	**1H (neutral)**	**AmH-3H⁺**	**AmH-1H⁺**	**ImH⁺**
NO_2	0.9123	0.9397	0.9162	0.9558	0.8940
COOH	0.8844	0.9201	0.8917	0.9363	0.8856
CHO	0.8789	0.9148	0.8851	0.9395	0.9301
Br	0.9028	0.8785	0.9089	0.8937	0.8721
Cl	0.9025	0.8766	0.9101	0.8919	0.8715
SH	0.8863	0.8900	0.8948	0.9035	0.8620
F	0.9026	0.8490	0.9115	0.8681	0.8611
H	0.8733	0.8790	0.8890	0.8944	0.8628
C_2H_5	0.8679	0.8690	0.8907	0.8827	0.8588
CH_3	0.8698	0.8667	0.8875	0.8828	0.8507
OCH_3	0.9043	0.8699	0.9109	0.8649	0.8795
OH	0.8938	0.8605	0.9030	0.8713	0.8766
NH_2	0.8887	0.8764	0.8915	0.8751	0.8722
Δ	0.0444	0.0907	0.0311	0.0909	0.0794
average	0.8898	0.8839	0.8993	0.8969	0.8752
SD	0.0145	0.0259	0.0110	0.0293	0.0203

Table S6 The slope coefficients of the HOMA vs cSAR(X) Equation 8, HOMA = a·cSAR(X) + b, with their standard errors s_a, ranges from a_{min} to a_{max} at the confidence level of 95%, and coefficients of determination R^2

**Tautomer**	**HOMA vs cSAR(X)**						
	**X**	***N***	***a***	s_a	a_{min}	a_{max}	R^2
3H	2	13	-0.586	0.106	-0.656	-0.516	0.735
1H		13	-0.566	0.111	-0.636	-0.496	0.701
AmH-3H$^+$		13	-0.426	0.093	-0.496	-0.356	0.654
AmH-1H$^+$		13	-0.446	0.091	-0.516	-0.376	0.684
ImH$^+$		13	-0.738	0.138	-0.808	-0.668	0.721
3H	5	13	-0.055	0.065	-0.125	0.015	0.060
1H		13	-0.326	0.047	-0.396	-0.256	0.816
AmH-3H$^+$		13	-0.022	0.064	-0.092	0.048	0.010
AmH-1H$^+$		13	0.475	0.087	-0.545	-0.405	0.732
ImH$^+$		13	0.023	0.098	-0.047	0.093	0.005
ImH$^+$		12[c]	-0.076	0.058	-0.146	-0.006	0.144

[c] Correlation for 12 substituents, the outlying data pair for CHO was rejected

Table S7 Estimated energies of hydrogen bonds E_{HB} (in kcal/mol) at bond critical points (BCPs) between the histamine derivatives and the amino acid residues of the receptor H_1.

**X**	**N1-H···O**	**N3-H···N**	**$_2$H^{α}N···H-O**
2-NO_2	10.09	6.64	14.30
2-Cl	6.45	9.13	16.79
H	6.26	11.62	17.17
2-Me	6.45	10.28	11.24
2-NH_2	6.26	11.62	14.11
5-NO_2	10.66	9.13	18.13
5-Cl	8.17	10.47	17.36
5-Me	5.11	11.81	16.79
5-NH_2	4.92	10.47	10.47

Table S8. The relative energies E_{rel} (in kcal/mol) for the histamine molecules and their C2 and C5 substituted derivatives in neutral and cationic forms.

	**C2**			**C5**		
	***E*rel(neutr)**	***E*rel(AmH+)**	***E*rel(ImH+)**	***E*rel(neutr)**	***E*rel(AmH+)**	***E*rel(ImH+)**
NO_2	-0.355	10.989	3.529	1.793	7.684	0.176
COOH	-0.179	10.505	-4.502	1.399	8.577	-3.926
CHO	-0.398	9.696	-0.876	-1.306	1.693	12.964
Br	0.155	10.872	-4.215	-0.656	9.050	-4.130
Cl	0.179	10.889	-3.070	-1.114	8.224	-3.760
SH	0.306	10.157	-6.465	0.683	10.574	-5.252
F	0.291	10.811	1.040	-3.310	4.352	-2.275
**H**	**0.288**	**11.143**	**-6.318**	**0.288**	**11.143**	**-6.318**
C_2H_5	0.392	10.709	-11.438	0.452	13.139	-10.011
CH_3	0.313	10.855	-9.976	0.574	12.628	-7.236
OCH_3	0.420	5.279	-10.247	-3.697	2.101	-7.218
OH	0.430	5.353	-6.407	-3.989	0.914	-7.411
NH_2	0.508	10.479	-9.413	-1.613	4.479	-9.053

$E_{\mathrm{rel}}(\mathrm{neutr}) = E_{\mathrm{3H-X}} - E_{\mathrm{1H-X}}$

$E_{\mathrm{rel}}(\mathrm{AmH+}) = E_{\mathrm{AmH-3H^+-X}} - E_{\mathrm{AmH-1H^+-X}}$

$E_{\mathrm{rel}}(\mathrm{ImH+}) = E_{\mathrm{ImH^+-X}} - E_{\mathrm{AmH-1H^+-X}}$

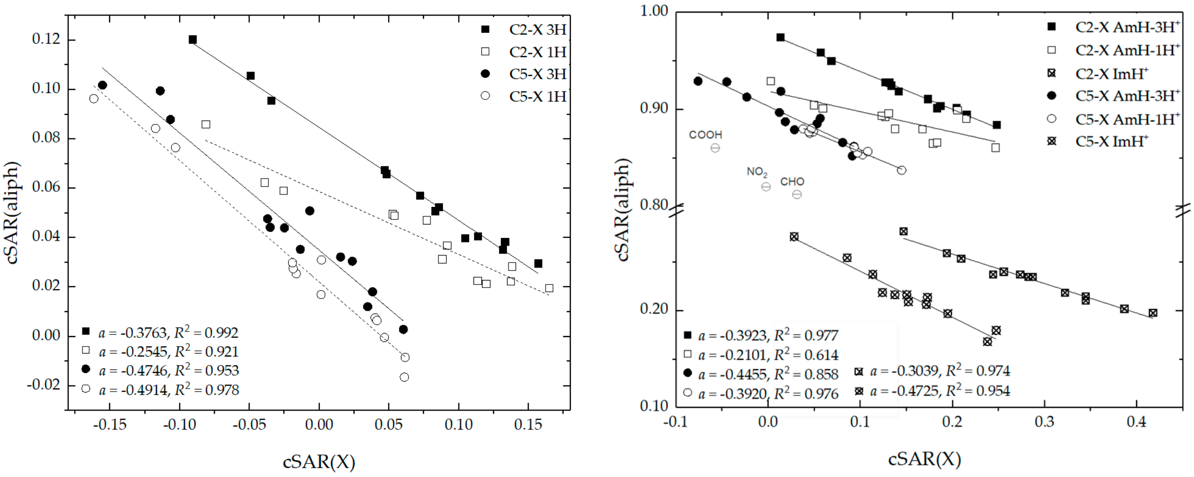

(**a**) (**b**)

Figure S1. Simple regression lines (Equation 8) fitted to the cSAR(aliph) vs. cSAR(X) sets of data for all histamine systems substituted at the C2 and C5 atoms in neutral (**a**), and cationic forms (**b**). All correlations are statistically significant (t.test$_{\alpha}$, $p < 0.05$).

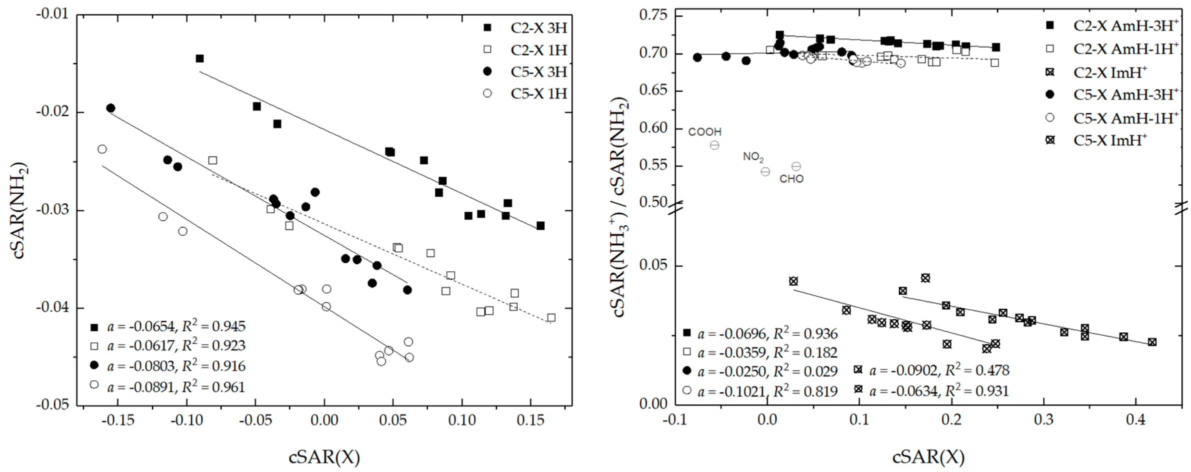

(**a**) (**b**)

Figure S2. Simple regression lines (Equation 8) fitted to the cSAR(NH_2) vs. cSAR(X) sets of data for all histamine systems substituted at the C2 and C5 atoms in neutral (**a**) and AmH$^+$ cationic (**b**) forms. The regression equation for the C5 substituted derivatives with the aliphatic chain coplanar to the ring was: cSAR(NH_2) = -0.0543·cSAR(X) + 0.6995, and R^2 = 0.2971.
